# Improving Chicken Responses to Glycoconjugate Vaccination Against *Campylobacter jejuni*

**DOI:** 10.3389/fmicb.2021.734526

**Published:** 2021-11-16

**Authors:** Harald Nothaft, Maria Elisa Perez-Muñoz, Tianfu Yang, Abarna V. M. Murugan, Michelle Miller, Daniel Kolarich, Graham S. Plastow, Jens Walter, Christine M. Szymanski

**Affiliations:** ^1^Department of Medical Microbiology and Immunology, University of Alberta, Edmonton, AB, Canada; ^2^Department of Agricultural, Food & Nutritional Science, University of Alberta, Edmonton, AB, Canada; ^3^Institute for Glycomics, Griffith University, Gold Coast Campus, Southport, QLD, Australia; ^4^Neogen, Edmonton, AB, Canada; ^5^ARC Centre of Excellence for Nanoscale BioPhotonics, Griffith University, Southport, QLD, Australia; ^6^Livestock Gentec, Edmonton, AB, Canada; ^7^Department of Microbiology and Complex Carbohydrate Research Center, University of Georgia, Athens, GA, United States

**Keywords:** vaccine, N-glycan, *Campylobacter*, immune response, fecal transplant

## Abstract

*Campylobacter jejuni* is a common cause of diarrheal disease worldwide. Human infection typically occurs through the ingestion of contaminated poultry products. We previously demonstrated that an attenuated *Escherichia coli* live vaccine strain expressing the *C. jejuni* N-glycan on its surface reduced the *Campylobacter* load in more than 50% of vaccinated leghorn and broiler birds to undetectable levels (responder birds), whereas the remainder of the animals was still colonized (non-responders). To understand the underlying mechanism, we conducted three vaccination and challenge studies using 135 broiler birds and found a similar responder/non-responder effect. Subsequent genome-wide association studies (GWAS), analyses of bird sex and levels of vaccine-induced IgY responses did not correlate with the responder versus non-responder phenotype. In contrast, antibodies isolated from responder birds displayed a higher *Campylobacter*-opsonophagocytic activity when compared to antisera from non-responder birds. No differences in the N-glycome of the sera could be detected, although minor changes in IgY glycosylation warrant further investigation. As reported before, the composition of the microbiota, particularly levels of OTU classified as *Clostridium* spp., *Ruminococcaceae* and *Lachnospiraceae* are associated with the response. Transplantation of the cecal microbiota of responder birds into new birds in combination with vaccination resulted in further increases in vaccine-induced antigen-specific IgY responses when compared to birds that did not receive microbiota transplants. Our work suggests that the IgY effector function and microbiota contribute to the efficacy of the *E. coli* live vaccine, information that could form the basis for the development of improved vaccines targeted at the elimination of *C. jejuni* from poultry.

## Introduction

*Campylobacter jejuni* is responsible for a majority of bacterial foodborne illness cases worldwide. It is also associated with the development of Guillain-Barré Syndrome (GBS), which is now the most common cause of paralysis since the near-eradication of polio (Willison et al., 2016). In addition, in low- and middle-income countries (LMICs), *Campylobacter* infection results in growth stunting and delayed cognitive development in young children (Kirkpatrick and Tribble, 2011; Kotloff et al., 2013; Lee et al., 2013; Platts-Mills et al., 2015; Amour et al., 2016; Dinh et al., 2016). Human infection mainly occurs through the consumption of contaminated chicken products and according to a recent CDC report, numbers of *C. jejuni* infections are increasing and causing a major burden to the healthcare system.^1^ Strategies to reduce *C. jejuni* in poultry include biosecurity measures; bacteriophages; bacteriocin treatment (Lin, 2009; Soro et al., 2020); antiadhesive strategies (Kelly et al., 2017); and inhibitors of biofilm formation, quorum sensing, secretion systems, or toxins (Kreling et al., 2020). In addition, post-harvest interventions, such as chemical treatment of meat, can decrease *C. jejuni* occurrence (Thames and Sukumaran, 2020). However, most of the approaches are still under development and the general use of antibiotics (although eliminated in their use as growth promoting substances) has resulted in increased antimicrobial resistance as an inevitable side effect among many microbes, including *Campylobacter* (Luangtongkum et al., 2009; Whitehouse et al., 2018; World Health Organization, 2017). As an alternative, vaccination of chickens could reduce *Campylobacter* at the source. Recent risk studies estimated that a 3-log10 reduction in broiler cecal concentrations of *C. jejuni* could reduce human infections by 58% (Hazards et al., 2020).

Promising vaccine candidates are bacterial surface carbohydrates that have been proven to be highly successful for human applications by inducing an effective and long-lasting immune response. Prominent examples are PREVNAR^®^ 13 and the *Haemophilus influenzae* type b capsular polysaccharide (Hib vaccine). Those vaccines are composed of capsular polysaccharides chemically conjugated to a protein/toxoid; however, their production for veterinary applications would be too costly. An alternative approach is the use of bioconjugates. Since the first discoveries that bacteria can glycosylate proteins, it has been demonstrated that both bacterial N- and O-linked protein glycosylation pathways can be transferred into a heterologous host (e.g., *Escherichia coli* or *Salmonella*) and be used to create heterologous glycoconjugates (Wacker et al., 2002, 2006; Feldman et al., 2005; Faridmoayer et al., 2008). Since then, experimental glycoconjugate vaccines have been engineered against *Brucella* (Iwashkiw et al., 2012), *Francisella tularensis* (Cuccui et al., 2013), *Escherichia coli* O121 (Wetter et al., 2013), *Shigella dysenteriae* and *Shigella flexneri* (Hatz et al., 2015; Riddle et al., 2016), *Burkholderia pseudomallei* (Garcia-Quintanilla et al., 2014), *Klebsiella* (Feldman et al., 2019), and *Streptococcus pneumoniae* (Harding et al., 2019).

For *Campylobacter*, the use of carbohydrate surface structures, such as lipooligosaccharides (LOS), associated with GBS-development, capsular polysaccharides (CPS) with at least 47 CPS different serotypes, or the variable O-glycans linked to flagella (formerly used for the heat-labile serotyping scheme), are not ideal. Further complexity is added by phase-variable expression of several of the underlying carbohydrate biosynthetic enzymes and variable modifications of CPS (e.g., with O-methyl phosphoramidate) and flagellar sugars (e.g., with acetamidino residues) to name a few (Guerry et al., 2002, 2012; St Michael et al., 2002; Karlyshev et al., 2005; Hendrixson, 2008; Mohawk et al., 2014). However, the N-glycan that is attached to at least 80 non-cytoplasmic proteins (Scott et al., 2011; Cain et al., 2019) and is encoded by the protein glycosylation pathway (*pgl*) is a common and invariable feature among *C. jejuni* isolates (Jervis et al., 2012; Nothaft et al., 2012). Moreover, this carbohydrate structure has been demonstrated to be immunogenic in mice, rabbits, and humans (Nothaft and Szymanski, 2010), and when expressed on the surface of *E. coli* (as a lipid A-core fusion) and administered as a live vaccine to chickens, induces an N-glycan-specific immune response and reduces the *Campylobacter* load by up to 10 logs (Nothaft et al., 2016, 2017). However, our previous studies have shown that while a subgroup of birds responded well to the vaccine leading to non-detectable levels of *C. jejuni* (responders), another subgroup remained colonized after vaccination and eventually showed *C. jejuni* colonization levels similar to unvaccinated birds (non-responders; Nothaft et al., 2016, 2017).

In this study, we performed additional chicken studies to gain a more mechanistic understanding about the factors that contribute to the responder versus non-responder effect in our vaccination and *C. jejuni* challenge model. We demonstrate that host genetics and the sex of the birds are non-factorial to the responder/non-responder phenotype. Although the level of IgY response does not correlate with the level of protection, sera from responder birds showed significantly higher bacterial killing activity compared to sera from non-responder birds which may be a consequence of the differences observed in IgY N-glycan core afucosylation. The microbial composition was also correlated with increased vaccine protection and birds transplanted with the cecal microbiota of responders showed improved immunological vaccine responsiveness. Our results therefore demonstrate that the vaccination-induced antigen responses and protection efficacy seem to be driven by N-glycan-specific antibody effector functions and the microbiota.

## Materials and Methods

### Vaccine Strain Construction

For stable expression of the *pgl* operon in the chromosome of avian pathogenic *E. coli* O:78(*aroA*) (Table 1), we created an operon that contains the minimal set of genes required for N-glycan expression. First, the *pglB* gene was removed from plasmid pACYC184*pgl* (p*pgl*); using p*pgl* as a template, one PCR product (*gne*-*pglH*) was amplified with oligonucleotides gne-SalI-F and pglJ-SbfI-R (Table 2) using the plasmid-borne *Sal*I site upstream of *gne* and introducing a unique *Sbf*I site after the stop codon of *pglH*. A second PCR product was generated with oligonucleotides pglA-SbfI-F and pglG-BamHI-R to amplify *pglA* to *pglG*, taking advantage of the *Bam*HI site on the plasmid downstream of *pglG* and introducing a *Sbf*I sequence upstream of *pglA*. Digested PCR products were inserted into plasmid pACYC184 cut with *Bam*HI and *Sal*I in a 3-arm ligation reaction, resulting in plasmid p*pgl*Δ*pglB*. For the insertion of the removable selection marker [kanamycin (*kan*) cassette flanked by FRT sites], a unique *Xho*I restriction site was generated between *pglK* and *pglH*: PCR products with oligonucleotide combinations pglK-XhoI-R, gne-SalI-F, and pglH-XhoI-F, pglJ-SbfI-R were used to amplify *gne*-*pglK* and *pglH* from template plasmid p*pgl* Δ*pglB*. *Sal*I-*Xho*I digested *gne*-*pglK* and *Xho*I-*Sbf*I digested *pglH* PCR products were inserted (3-arm ligation reaction) into *Sal*I-*Sbf*I digested plasmid p*pgl*Δ*pglB* resulting in plasmid p*pgl*Δ*pglB-Xho*I. Next, the *kan* cassette flanked by FRT sites was PCR amplified from plasmid pKD4 using oligonucleotides P1-pKD4-XhoI and P2-star-pKD4-XhoI introducing *Xho*I sites in the 3' and 5'-prime ends. The obtained product was digested with *Xho*I and inserted into the *Xho*I-linearized p*pgl*Δ*pglB-Xho*I plasmid. One positive candidate plasmid where the *kan* cassette is transcribed in the same orientation as the *pgl* genes was named p*pgl*-kan. To remove the upstream region of *gne* and genes *pglC* to *pglG*, a PCR product encompassing *gne*-*pglK-kan-pglHIJA* was generated with oligonucleotides gne-BamHI-F1 and pglA-BamHI-R and inserted into *Bam*HI digested plasmid pACYC184 (Supplementary Figure S1A).

**Table 1:** Oligonucleotides.

Oligo	Sequence 5' to 3'	Use
P1-pKD4-XhoI	ATATATCTCGAGTGTAGGCTGGAGCTGCTTCG, *Xho*I	PCR FRT-kan-FRT
P2-star-pKD4-XhoI	TATATACTCGAGCATATGAATATCCTCCTTAG, *Xho*I	PCR FRT-kan-FRT
O78pol-BamHI-F	ATATGGATCCATTGATCTCTATTATCGTATTATTCCAC, *Bam*HI	PCR wzy^O78^
O78pol-EcoRI-R	ATATGAATTCGTTCGTAATGTGTCATTTCTCCATCGACAC, *Eco*RI	PCR wzy^O78^
O78 invers BglII-F	AATTAGATCTTTCCATTCGTTATTTGATAGC, *Bgl*II	Inverse PCR
O78 invers BglII-R	TTAAAGATCTAAGCGAATAACGCAGCAGCAAC, *Bgl*II	Inverse PCR
gne-SalI-F	ATCGGTCGACGCTCTCCCTTATGCG, *Sal*I	PCR gne to pglJ
pglJ-SbfI-R	AACCACTTAACGTTTAACCTGCAGGATATAACGTGCAATTTTTACTTTATCAAAAGC, *Sbf*I	PCR gne to pglJ
pglA-SbfI-F	AATTGCAATTTATATCCTGCAGGTTAAAATTTAAGGGTTGAAAATGAGAATAGG, *Sbf*I	PCR pglA to pglG
pglG-BamHI-R	TCCTGTGGATCCCCCGGGCTGCAGGAATTCGATC, *Bam*HI	PCR pglA to pglG
pglH-XhoI-F	ATATCTCGAGAGCTTAAAGAGGAGAAATGATGAAAATAAGC, *Xho*I	PCR pglH
pglK-XhoI-R	ATATCTCGAGATCATTTCTCCTCTTTAAGCTTACC, *Xho*I	PCR gne to pglK
gne-BamHI-F1	AATGGATCCAGTTTTTGCAAAAGCTTGTG, *Bam*HI	PCR gne to pglA
pglA-BamHI-R	AATGGATCCTCATACATTCTTAATTACCC, *Bam*HI	PCR gne to pglA
O78-outside-F	TCATAATATCATAACAGGGGTCATGAGC	PCR, confirm integration
O78-outside-R	TTGTGATTGTATAACCCAGCTCTTTATAC	PCR, confirm integration
pglA-F	AAATGCTGCCCAAGATGCTTTACAATACG	PCR, confirm integration
gne-R	TTAAAGTATGAGAACCTATATAACCTGC	PCR, confirm integration

*Restriction sites are underlined; restriction enzymes (if applicable) are indicated*.

**Table 2:** Strains and plasmids.

Strain	Genotype or relevant markers	Reference
***Campylobacter***		
*C. jejuni* 81-176	Clinical isolate, wild-type strain	Korlath et al., 1985
***E. coli***		
DH5α	F^−^ *endA1 glnV44 thi-1 recA1 relA1 gyrA96 deoR nupG purB20* φ80d*lacZ*ΔM15 Δ(*lacZYA-argF*)U169, hsdR17(*r_K_*^−^*m_K_*^+^), λ^−^	Invitrogen
APEC O:78(*aroA*)	Avian pathogenic *E. coli*, serotype O:78, mutation in *aroA*	Poulvac^®^ *E. coli*
APEC O:78(*aroA*)/*wzy*::*pgl::kan*	APEC O:78(*aroA*) with *pgl* (*gne*-*pglK*-FRT-*kan*-FRT-*pglHIJA*) inserted into *wzy* locus, Kan^R^	This study
Plasmid		
pBluescript II KS(+)	Cloning vector, pUC ori, Amp^R^	Agilent
pACYC184	Cloning vector, P15A ori, Cm^R^, Tet^R^	Chang and Cohen, 1978
pACYC184*_pgl_* (p*pgl*)	Plasmid pACYC184 containing the 16kb *C. jejuni pgl* locus, Cm^R^	Wacker et al., 2002
p*pgl* Δ*pglB*	p*pgl* with a deletion of *pglB*, Cm^R^	This study
p*pgl* Δ*pglB-Xho*I	p*pgl* Δ*pglB* with *Xho*I site between *pglK* and *pglH*, Cm^R^	This study
p*pgl*-Kan	p*pgl* Δ*pglB*, *Xho*I with Kan cassette (flanked by FRT sites) inserted between *pglK* and *pglH* (*via Xho*I), Cm^R^	This study
p*pgl*gneK-kan-HIJA	PCR product containing *gne*-*pglK*-FRT-*kan*-FRT-*pglHIJA* inserted into pACYC184, Cm^R^	This study
pBKS-wzy^O78^	*wzy* (*rfc*) of APEC O78 inserted into pBluescript II KS(+), Amp^R^	This study
pBKS-wzy^O78^, *Bgl*II	pBKS-*wzy*^O78^ with *Bgl*II sites inserted into *wzy*, by inverse PCR	This study
pBKS-wzyO78-pgl-kanFRT	pBKS-*wzy*^O78^ with PCR product *gne*-*pglK*-FRT-*kan*-FRT-*pglHIJA* inserted into *Bgl*II site of wzy^O78^, Amp^R^	This study
pKD4	FRT flanked kan cassette, template plasmid for PCR, Kan^R^, Amp^R^	Datsenko and Wanner, 2000
pKD46	λ Red recombinase (γ, β, and exo from λ phage), *ara*C-*Para*B, Amp^R^	Datsenko and Wanner, 2000

The targeted insertion locus the *wzy* (*rfc*) gene of strain APEC O:78 was amplified from chromosomal DNA with oligonucleotides O78pol-BamHI-F and O78pol-EcoRI-R (Table 2) and ligated into *Bam*HI-*Eco*RI digested pBluescript II KS(+) (Table 1). For the insertion of the minimal *kan* cassette containing *pgl* operon, a unique *Bam*HI compatible *Bgl*II site was generated by inverse PCR with oligonucleotides O78 inverse BglII-F and O78 inverse BglII-R using plasmid pBSK-*wzy*O78 as template. The obtained construct after re-ligation of the inverse PCR product was digested with *BglI*I and PCR amplified *gne*-*pglK-kan-pglHIJA* was inserted. After ligation and selection for Kan and Amp resistant colonies, one candidate in which the *pgl* genes are transcribed in the same orientation as the *wzy*^O78^ gene was (after linearization with *Eco*RV) used for integration into APEC O:78(*aroA*) following the method of Datsenko and Wanner (2000; Supplementary Figure S1B). The correct insertion of the construct was verified by PCR with *pgl*-gene-specific oligonucleotides and primers that hybridize outside of the recombination event. Expression of the lipid A-core N-glycan in the vaccine strain, APEC O:78(*aroA*)/*wzy*::*pgl::kan* (Table 2), was verified by Western blotting with *Cj*-N-glycan-specific (R1) antiserum as described (Nothaft et al., 2016; Supplementary Figure S1C).

### Chicken Vaccination and Challenge Studies

#### Experiment A, B, and C

Three independent but identical experiments (Experiment A, B, and C) were conducted. Experiment A took place in Jan/Feb, B in March/April, and C in May/June. For each replicate, the vaccine groups consisted of 45 birds; the negative control groups (not vaccinated and not challenged) of five birds, and the positive control (not vaccinated but challenged) groups of 10 birds. For each experiment, commercial broiler birds (Ross 308; Avigen) were obtained on the day of hatching from Lilydale (Edmonton, AB) and transported by the Poultry Research Facility, University of Alberta. Upon arrival at the Science Animal Support Services facility, birds were tagged randomly. Groups of four birds were placed in cages with a ventilated filter cage top and false bottoms and were bedded with autoclaved aspen chips. If birds/groups were not a multiple of 4, remaining birds were stocked in groups of three birds/cage. Cages were placed on metal shelves in a temperature and humidity-controlled room and water and feed (Laboratory Chick Diet S-G 5065*, Teklad Diets, Madison WI) was given *ad libitum*. Bedding was replaced every 2–3days. After the first vaccination (day 7), birds initially stocked at four bird/cage were separated in two birds per cage and at the day of the second vaccination (day 21), all birds were individually housed (one bird/cage) until the day of euthanasia. To verify and test the APEC O:78-based N-glycan vaccine, an established 35-day vaccination and challenge protocol were applied as described (Nothaft et al., 2016, 2017). In particular, birds were randomly tested (cloacal swabs) for the presence of *Campylobacter* on the day of hatch (day 0). Vaccination with the *E. coli* live vaccine was done by orally gavaging each bird with 1×10^8^ live APEC O:78(*aroA*)/*wzy*::*pgl::kan* cells in PBS on days 7 and 21. Control groups were gavaged with PBS only. Birds were sampled for *E. coli* vaccine persistence by cloacal swabbing at various time points and plated into media selective for the live vaccine strain. Birds were challenged (orally gavaged) on day 28 with either PBS (negative control) or with PBS containing 1×10^6^CFU of *C. jejuni* 81-176. Then, 7-days post-challenge, birds were euthanized according to the approved guidelines of the Canadian Council for Animal Care. *Campylobacter* colonization levels in poultry ceca were determined by resuspending individual cecal contents in an equal volume (v/w) of PBS, 40% glycerol followed by preparing and plating 25-μl aliquots of 10-fold serial dilutions (made in PBS) on *Campylobacter*-selective Karmali agar. Differences in bird colonization were analyzed for significance using Fisher’s exact tests. If applicable, the second cecum was used for subsequent microbiota sequencing (as outlined below).

Blood samples on day 0 and prior to challenge were collected from the wing vein, or by heart puncture on the day of euthanasia in heparin-coated tubes. Sera were prepared as described (Nothaft et al., 2016). Cecal samples (initially 1:1 diluted in PBS, 40% glycerol) and stored at −80°C were thawed on ice, centrifuged (10min, 20,500×*g*, 4°C) and each supernatant was transferred to a fresh tube. For cecal sample ELISA (IgY and IgA), equal amounts of sample from each PBS, *Cj*, and responder and non-responder birds from Experiments A, B, and C were pooled. Serum IgY and day 35 cecal IgY and IgA levels were determined in a 96-well ELISA plate format (Nothaft et al., 2016). Briefly, 96-well ELISA plates (Maxisorb, Thermo Fisher) were coated with a BSA-N-glycan conjugate (capture antigen, 500ng/well) for 18h at 4°C. Unbound capture antigen was removed and wells were blocked at room temperature for 1h with 100μl of 5% skim milk in PBS-T with shaking. Removal of blocking solution was followed by addition of antibody sera (individual or pooled as described below from day 1, 28, or day 35) or cecal content supernatants (from day 35) diluted 1:10 in PBS-T with 1% skim milk. After incubation for 1h at RT and three washing steps with 100μl of PBS-T, 100μl of anti-chicken IgY-AP or anti-chicken IgA-AP (diluted 1:500 in PBS-T with 1% skim milk) served as the secondary antibodies (for 1h at RT) followed by four washing steps with 100μl of PBS-T. Plates were developed using pNPP (Thermo Fisher) according to instructions provided by the manufacturer and scanned at OD_405_ in a plate reader. Differences in the IgY and IgA levels in the various groups were investigated for significance using two-tailed *t*-tests. Sera and cecal sample supernatants were stored at 4°C until further use, whole blood for DNA extraction/genotyping (GWAS) was stored as outlined below.

#### Persister Challenge

Single *Campylobacter* colonies that appeared on Karmali plates after plating of cecal content from vaccinated and challenged birds from Experiments A, B, and C (non-responders, 10 colonies per plate, phenotypically white/slightly pink, mix of pinpoint, and non-pinpoint colonies) were collected in 96-well plates (with 200-μl MH medium in each well) and incubated until stationary phase (OD_600_ of approx. 1.2–1.4). Equal amounts (50μl) from each well were pooled, centrifuged for 5min at 8,000×*g*, 4°C, and resuspended and adjusted to 3×10^6^CFU/ml (3×10^6^CFU/0.3ml challenge dose) using ice-cold PBS. Remaining cells were supplemented with glycerol to a final concentration of 20%, aliquoted in 96-well plates, and stored at −80°C. Housing, vaccination/challenge, sample collection, and processing were done as described above.

#### Cecal Microbiota Transplantation

Microbial communities isolated from non-colonized (responder) birds were characterized for their ability to further reduce *Campylobacter* in poultry.

First, we analyzed the effect of antibiotics with the intent to deplete the gut microbiota. To do so, birds received antibiotics with the drinking water [vancomycin (500mg/L), metronidazole (800mg/L), ampicillin (1.5g/L), and ciprofloxacin (300mg/L)] on days 3–5; then, water was switched to normal drinking water on day 6. Birds were euthanized on day 7 and the composition of the microbiota was determined as described below. For CMT experiments, birds received a similar antibiotic treatment before oral vaccination/bacterial community feeding on day 7. Microbial communities were collected from the cecum of all responder birds by resuspending the second cecum (collected from Experiments A, B, and C) in 1-ml ice-cold, filter-sterilized PBS per gram cecal weight and equal volumes of each suspension were pooled. After allowing insoluble material to settle for 5min (on ice), bacteria were aliquoted, supplemented with glycerol to a final concentration of 20%, and stored at −80°C until further use. For reconstitution experiments, aliquots were thawed on ice, DAPI-stained, quantified microscopically (bacterial cell count), and set to the desired concentration (1×10^8^cells) with ice-cold, filter-sterilized 1×PBS. Birds were orally gavaged with the *E. coli* live vaccine in combination with an equal amount of microbial community cells (1×10^8^cells) followed by challenge with *C. jejuni* (using the above-described experimental conditions, vaccination, and challenge regimen). *C. jejuni* colonization levels, the N-glycan-specific IgY responses, vaccine persistence, the composition of the microbiota in selected birds, and statistical analyses were performed as described above.

### Genome-Wide Association Study

#### Sample Preparation and Analyses

DNA for genome-wide association study (GWAS) was isolated as follows: a fraction of each blood sample from day 35 birds was supplemented with EDTA (to a final concentration of 10mM) to prevent agglutination. Samples were frozen immediately and were kept at −80°C until further use. Then, 10μl from each sample was used to isolate chromosomal DNA with the DNeasy Blood & Tissue Kit (Qiagen) following the manufacturer’s protocol. DNA was dissolved in nuclease free water and samples were normalized to 50ng/μl or 750ng total. In total, 134 chickens (88 responders and 46 non-responders) from Experiments A, B, and C were genotyped using the Affymetrix Axiom 580k (Thermo Fisher) assay (Kranis et al., 2013). In total, 580,961 SNPs were genotyped for each chicken. SNPs that were unmapped (*n*=7,316), unlocalized and unplaced (*n*=653), or located on allosomes (*n*=26,520) were removed from analysis. For the remaining 546,472 SNPs located on autosomes, we further removed SNPs that met any of the three criteria: (1) minor allele frequency (MAF)<5%, (2) call rate<90%, or (3) have Hardy-Weinberg equilibrium exact test values of *p* below 10^−10^. In total, 475,058 SNPs passed the quality control and were used in the analysis.

#### Population Stratification

The population structure of the 134 chickens was assessed based on their genotype data. The pairwise genetic distance between chickens were estimated using Plink 1.9 (Chang et al., 2015). The population structure was visualized using multidimensional scaling (MDS). We also conducted a principal component analysis (PCA) to assess the effect of potential population stratification.

#### Association Analysis

Association test was conducted using Plink 1.9 (Chang et al., 2015). Fisher’s exact test was used to test the difference in genotypic frequencies between responders and non-responders. The result was adjusted for multiple testing using false discovery rate (FDR).

### Microbiota Analyses

Total DNA was extracted from chicken cecum samples as described (Nothaft et al., 2016). Composition of the bacterial community in cecal samples was characterized using 16S rRNA gene amplicon sequencing. PCR targeting the V6-V8 region of the 16S rRNA gene with primers 926F (5'-AAACTYAAAKGAATWGRCGG-3') and 1392R (5'-ACGGGCGGTGWGTRC-3'), and subsequent amplicon sequencing (Illumina MiSeq platform producing 300-bp paired-end sequences) was performed. Amplicon sequencing produced a total of 27,819,932 raw sequences (average=137,035; minimum=76,068 and maximum=208,755). For each sequencing run, raw reads were trimmed to 270 bases long using FASTX-toolkit (v.0.0.14).^2^ R1 and R2 ends were quality filtered and paired using the merge-illumina-pairs application from Illumina utils v.2.0.1 (Eren et al., 2013). Sequences that did not meet the quality criteria (value of *p* of 0.03, enforced Q30 check, perfect matching to primers, and no ambiguous nucleotides allowed) were discarded. After merging and quality control, a total 13,124,620 sequences were obtained (average=71,376; minimum=10,795 and maximum=129,875). To make the dataset manageable, samples were randomly subsampled to obtain 20,000 reads using mother v.1.39.2 (Schloss et al., 2009). All reads were kept for samples that had less than 20,000 sequences (total of three samples; range of 10,795–17,532 sequences). Subsequently, reads were compiled and dereplicated, singletons were discarded, and chimeras removed using usearch v.10 (Edgar, 2013). After dereplication and chimera removal, all reads from all sequencing runs were combined and OTUs were clustered at 98% identity, representative sequences for each OTU were selected, and the OTU table was generated also using usearch v.10 (Edgar, 2013). Non-chimeric sequences were binned by sample and submitted to Ribosomal Database Project Classifier (Wang et al., 2007) for taxonomic assignment from phyla to genera. OTUs were assigned taxonomy using Silva database (Westram et al., 2011) and sequence identity was confirmed using NCBI blastn (Altschul et al., 1990), EZ biocloud (Yoon et al., 2017), and Ribosomal Database Project Seqmatch (Michigan State University, 2016). Counts were transformed to relative abundance. Taxa with a mean relative abundance less than 0.01% were removed from the dataset prior to statistical analyses. Diversity analyses were performed using Qiime v.1.9.1 (Caporaso et al., 2010).

To determine differences between responders and non-responders for the different taxonomic levels, multiple *t*-tests followed by FDR test (determined using the two-stage linear step-up procedure of Benjamini, Krieger, and Yekutieli) were performed using GraphPad Prism version 8, GraphPad Software, La Jolla, California, United States.^3^ To determine significance between responders and non-responders for diversity metrics, Mann-Whitney tests were performed also using GraphPad Prism version 8. To determine significance of non-metric multidimensional scaling based on Bray-Curtis distances, PERMANOVA test was performed using the vegan v2.5-7 package (Oksanen et al., 2017) in R version 3.5.1 (R-Core-Team, 2018). The vegan package was also used to determine the goodness of fit statistic (squared correlation coefficient *r*^2^) of unconstrained ordination through the application of the envfit function. PCA plots were generated using Euclidean distances using basic statistic functions in R. Random forest analysis were performed using the randomForest v4.6-14 package in R. Abundance values were transformed to Z-scores prior to analyses to normalize data. To decrease the error rate of the forest, the best mtry (random variables used in each tree) was calculated and used to fine-tune the forest. ROC plots and AUC were determined using the RCOR v1.0 package in R (Sing et al., 2005).

### Antibody Characterization

#### Opsonophagocytosis Assay

For opsonophagocytosis assays, *C. jejuni* cells were incubated with fresh heparinized chicken blood and either naïve chicken serum or pooled chicken serum (from responder and non-responder birds) according to the method previously described by Goyette-Desjardins et al. (2016) with modifications (Wenzel et al., 2020). To prepare the bacterial cells, *C. jejuni* 81-176 was grown on BHI plates. On the day of blood collection, *C. jejuni* cells were harvested in PBS, pelleted by centrifugation at 13,000×*g* for 5min, and washed twice with PBS. Washed cells were suspended to 3×10^5^ cells per ml in RPMI 1640 media supplemented with 5% heat inactivated chicken serum, 10 mM HEPES, 2 mML-glutamine, and 50 μm β-mercaptoethanol. Fresh chicken blood (collected in a heparin-coated tube to prevent coagulation) was diluted 1/3 in above-described supplemented RPMI 1640 and used in reactions with pooled sera from either responder or non-responder birds. To do so, each of the diluted blood samples (50μl) was combined with 40μl of either naïve (PBS negative control group) chicken serum or the pooled responder and non-responder sera, followed by addition of 10μl of the *C. jejuni* cell suspension, resulting in an approximate MOI of 0.015 based on 3×10^3^ bacterial cells in the reaction and a calculated content of 1.9×10^5^ leukocytes based on the literature values of leukocytes in the blood of broiler chickens (Orawan and Aengwanich, 2007). Tubes were subsequently placed in a 5% CO_2_ incubator at 37°C for 2h and samples were mixed every 10–15min (by inversion). Aliquots of 10-fold serial dilutions were plated in triplicate on BHI agar with *Campylobacter*-selective supplement and incubated under microaerobic conditions for 24h. Bacterial killing was calculated as a fraction (% bacteria) of the number of CFU obtained in the control assay that did not contain N-glycan-specific (pooled serum), but serum from naïve (PBS negative control group) chickens. Statistical significance between samples was determined by two-tailed *t*-tests.

#### Analysis of the Chicken Serum and the IgY Glycome

##### Serum/IgY N-Glycomics Using PGC LC-ESI MS/MS

N-glycans from total chicken serum were released using PNGase F and reduced as described previously (Jensen et al., 2012). In short, serum samples (approximately 35μg protein) were immobilized into PVDF membrane and visualized using Direct-blue. The N-glycans were released using PNGase F and reduced. They were separated using Porous Graphitised Carbon Nano Liquid Chromatography [nanoFlow column containing 5-μm Porous Graphitised Carbon (Hypercarb, PGC, Thermo Fischer Scientific)] and analyzed by Electrospray tandem mass spectrometry (PGC-nano LC-ESI MS/MS) on the Captive Spray Ionization Source (Amazon Ion Trap, Bruker) with 1.2kV capillary voltage and 150°C as dry gas temperature (Moginger et al., 2018). The acquired mass spectra were analyzed manually using Bruker Compass Data Analysis 4.2 (Bruker GmbH, Bremen, Germany). The corresponding glycan compositions were predicted using Glycomod^4^ and the structures verified by manual validation using their individual MS/MS spectra and their corresponding retention times on the PGC column as described previously (Kolarich et al., 2015; Hinneburg et al., 2017). Quantitation of data obtained using nanoPGC LC ESI MS/MS was performed in Small molecule mode in the open access software, Skyline (Adams et al., 2020). Statistically significant differences were calculated using two-way ANOVA.

##### High-Throughput Analysis Serum N-Glycans Using MALDI-TOF MS

N-glycans were released in-solution by PNGase F and derivatized by ethyl esterification using EDC (N-(3-Dimethylaminopropyl)-N'-ethylcarbodiimide) and HOBt (Hydroxybenzotriazole hydrate) in 100% ethanol as described previously with modifications (Reiding et al., 2014). Briefly, the derivatized N-glycans were purified using Cotton HILIC columns (little 2–3mm diameter cotton balls instead of pipette tips fitted with cotton HILIC) in a 96-well plate. The purified glycans were mixed with equal volume of HCCA matrix prepared with 85% acetonitrile and 0.1% TFA and spotted into 384 MALDI Ground steel plates.

The sample spots were analyzed on a Bruker RapifleX MALDI-TOF mass spectrometer (Bruker, GmbH, Bremen, Germany) in Reflectron positive mode (Reiding et al., 2014). Each sample spot was analyzed using an Automatic method: 20,000 shots were accumulated within the mass range 800–4,000m/z using random walking pattern with 200 laser shots fired per raster for a diameter restricted to 2,000μm. The calibrated spectral files for each spot were exported into text format (x,y) using FlexAnalysis version 4.0, build 14 (Bruker). The exported files were baseline corrected and the detected glycan compositions were extracted as a batch operation using the open-source software called “Massytools” (Jansen et al., 2015). The analyzed data were curated and plotted in GraphPad Prism. Statistical significance was determined using two-way ANOVA.

##### Enrichment of Chicken IgY

Chicken IgY was enriched using Ligatrap Chicken IgY Purification Resin (LigaTrap Technologies). Then, 5μl of total chicken serum was used as starting material and the procedure was followed as per the manufacturer’s instructions. The presence of IgY was confirmed by SDS-PAGE and proteomics using C18 nano LC coupled to a Thermo Orbitrap Fusion MS.

## Results

### Vaccination Significantly Reduces the *Campylobacter* Load in Broiler Chickens

**Figure 1:**
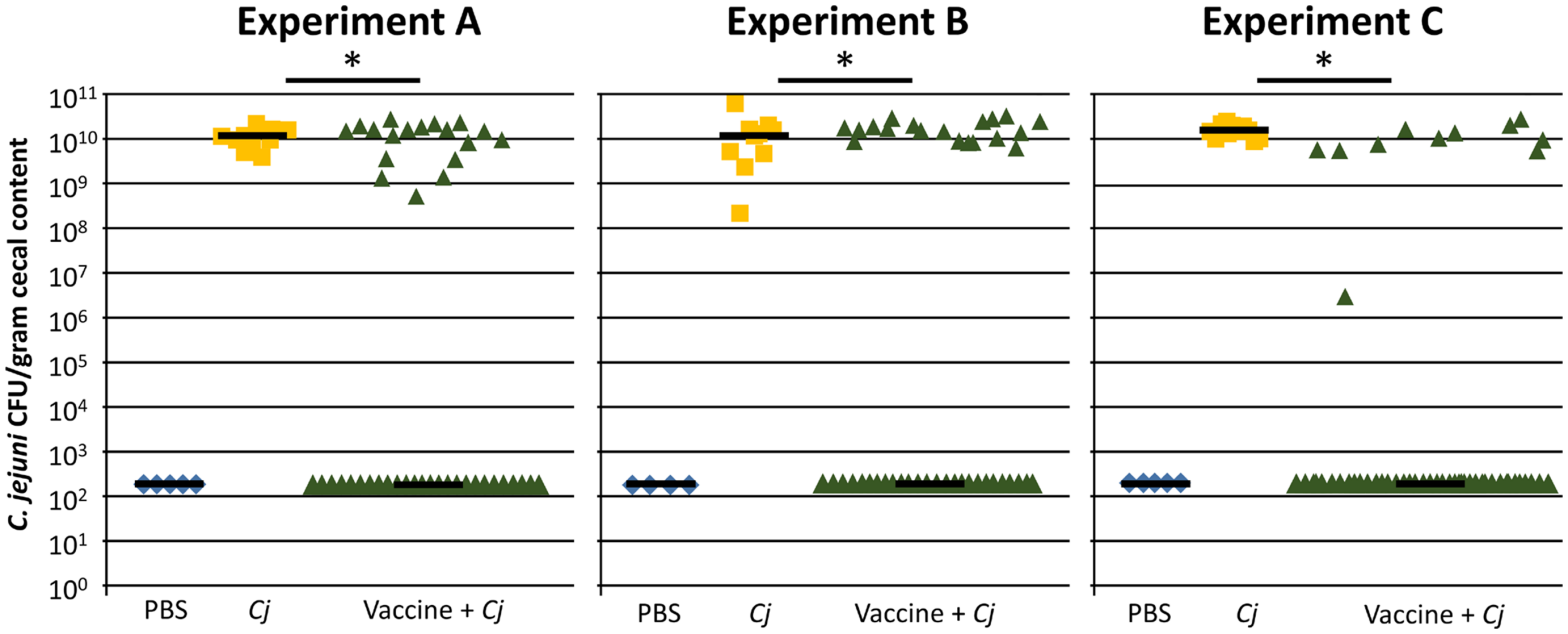
The APEC O78-based *C. jejuni* vaccine significantly reduces *C. jejuni* colonization in broilers. Results from three independent *E. coli*-based vaccination and *C. jejuni* strain 81-176 challenge studies are shown. Birds in the negative control group (PBS, Experiment A, B, C; *n*=5, 4, 5) were non-vaccinated and non-challenged; birds in the positive control group (*Cj*, *n*=10, 10, 10) were non-vaccinated but challenged with 1×10^6^CFU of *C. jejuni* wild type on day 28. Birds in the treatment groups (Vaccine + *Cj*, *n*=44, 45, 46) were orally gavaged on days 7 and 21 with 1×10^8^CFU of the attenuated APEC O78-based *C. jejuni* vaccine and challenged with 1×10^6^CFU of *C. jejuni* wild type on day 28. Statistically significant differences (Fisher’s exact test, *p*<0.001) are indicated by an asterisk. PBS, Phosphate-buffered saline; *Cj*, *Campylobacter jejuni*.

Three independent replicates of a vaccination and challenge experiment (Experiments A, B, and C) were conducted to verify the efficacy of the avian pathogenic *E. coli* (APEC) O78-based vaccine strain that expresses the *C. jejuni* N-glycan on its surface (Figure 1 and Supplementary Figure S1). Each replicate consisted of a vaccine group (vaccinated and challenged), a positive control group (not vaccinated, challenged), and a negative control group (not vaccinated, not challenged). In all three experiments, the *C. jejuni* colonization levels in cecal samples, taken on day 35, showed a similar bimodal distribution that has been observed in previous experiments (Nothaft et al., 2016, 2017). A statistically significant proportion (determined by Fisher’s exact test, *p*<0.001) of birds (averaging 65%) were found to be fully protected after vaccination (= responders), while an average of 35% of birds was still colonized after *C. jejuni* challenge (= non-responders) as determined by colony counts obtained after serial diluting the cecal contents of each bird and culturing on selective plates for 48 h (Figure 1) and subsequently confirmed by 16S sequencing (see below). In particular, in Experiment A, 26 out of 44 (= 60%); in Experiment B, 26 out of 46 (= 57%), and in Experiment C, 36 out of 46 (= 78%) had non-detectable levels of *C. jejuni*, by both methods, in their ceca. It is worth mentioning that minor deviations from the original group sizes were due to unrelated mortalities or morbidities (e.g., birds with splayed leg/unable to move were euthanized according to the approved animal protocol). In contrast, all birds (100%) in the positive control group were colonized with *Campylobacter* (statistical mean of 1.1×10^10^CFU/gram cecal content) and no *Campylobacter* was detected in birds in the negative control groups. The live vaccine strain persisted in at least 60% of the birds up to day 10 (3days after the first vaccine dose) and in 12–40% 4days after of the second vaccine dose (day 25; Supplementary Figure S2). In experiments A and B, we could still detect the live vaccine strain in nine (out of 44) and four (out of 45) of the vaccinated birds at the day of euthanasia (day 35) and in experiment C, the vaccine strain could not be detected on day 35. However, no correlation between vaccine persistence and the responder/non-responder effect could be established (Fisher’s exact test, *p*=0.8).

### Vaccination Induces an N-Glycan-Specific Immune Response

**Figure 2:**
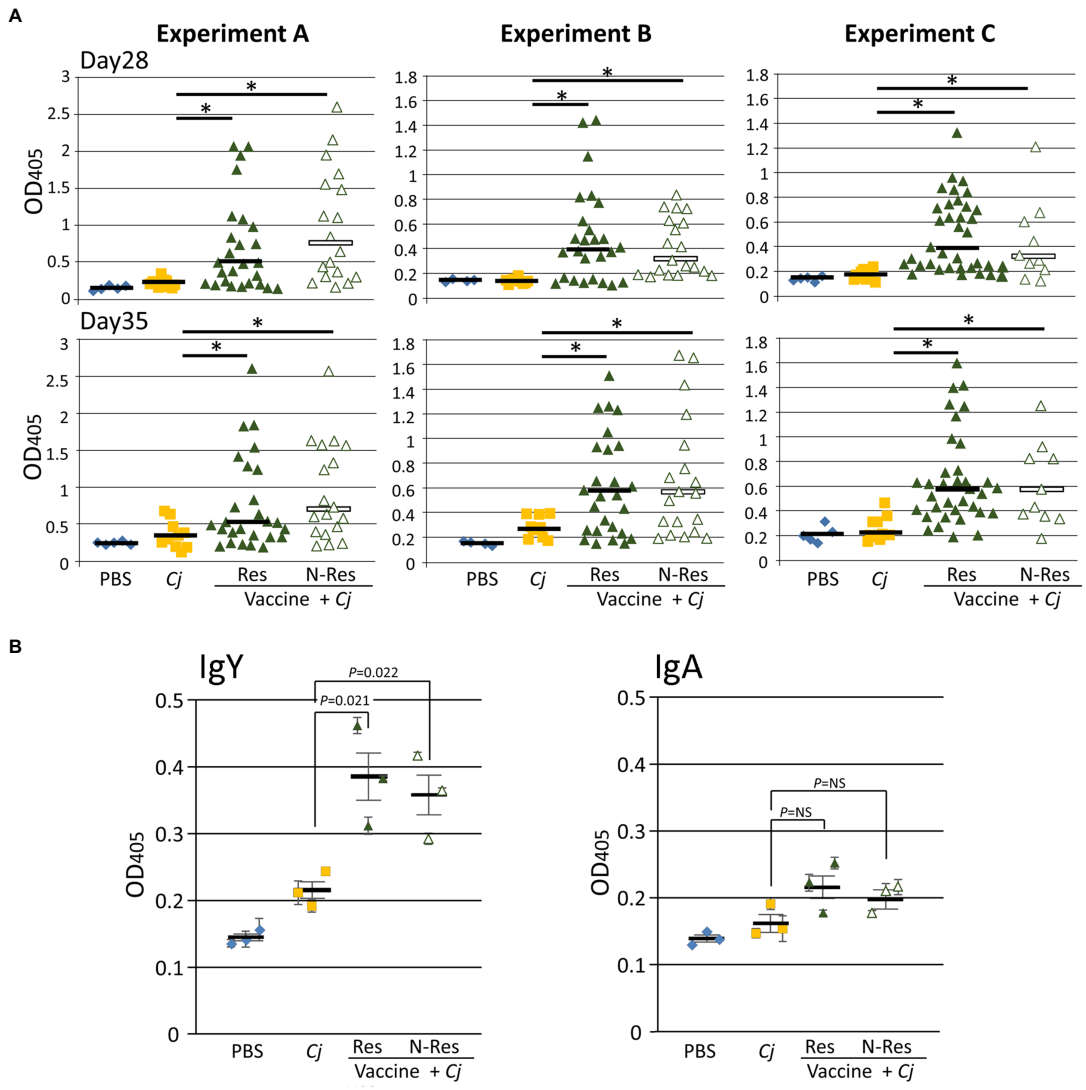
The N-glycan-specific immune responses. **(A)** ELISA results comparing chicken sera from bleeds taken prior to *C. jejuni* challenge (day 28) and at day 35. Each point represents the N-glycan-specific antibody response measured at OD_450_ for each individual chicken from the indicated group. For the vaccine + *Cj* groups, responders (Res) and non-responders (N-Res) are shown as individual subgroups; no significant differences between the two subgroups (two-tailed *t*-test, *p*>0.05) were found. No N-glycan-specific antibodies were detected in sera of blood samples taken on day 1. **(B)** ELISA results comparing N-glycan-specific IgY and IgA levels (as indicated) in individually pooled cecal contents from each group. Each dot in the respective group represents one study (Experiment A, B, or C=biological replicate) with measurements carried out in triplicate and error bars representing the standard error of the mean for each replicate. Bars represent the median for each group/subgroup and error bars in **(B)** indicate the standard error of the mean (SEM) for the biological replicates. Statistically significant differences (two-tailed *t*-test, *p*<0.05) are either indicated by an asterisk or the value of *p* is provided for the specific comparison, NS=not significant. PBS, Phosphate-buffered saline; *Cj*, *Campylobacter jejuni*.

First, we determined the N-glycan-specific immune responses using our established ELISA format (Nothaft et al., 2016). In all three vaccination experiments, N-glycan-specific IgY responses were observed in sera prepared from blood taken 1day prior to challenge (day 27) as well as in the sera prepared from blood taken on day 35 (Figure 2A). In addition, we were able to detect N-glycan-specific IgY and IgA in cecal content supernatants individually pooled from vaccinated and challenged birds (Figure 2B). Whereas cecal IgY levels were significantly increased compared to the control groups, the observed increase in N-glycan-specific IgA levels in the vaccinated and challenged groups was not statistically significant when compared to the control groups. However, and as seen in our previous studies (Nothaft et al., 2016, 2017), no correlation between the antibody titers and the level of protection could be established and no statistically significant difference was observed in the IgY level between responders and non-responders.

### Vaccine-Induced Antibodies Possess Opsonophagocytosis Activity

**Figure 3:**
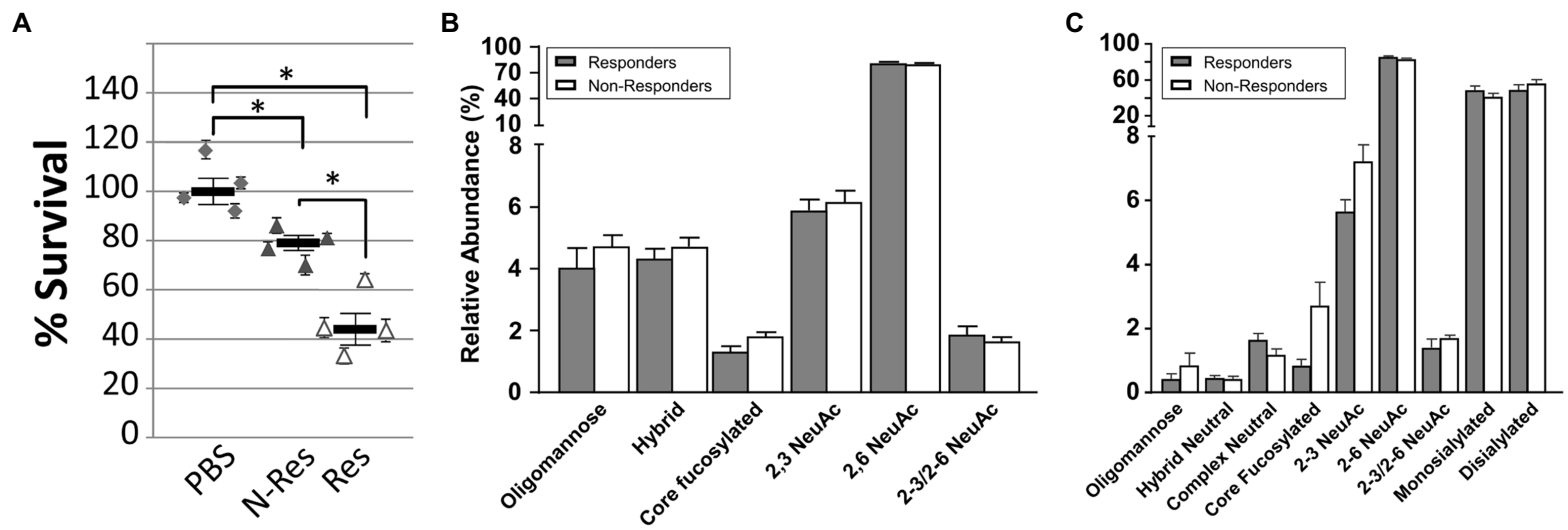
Antibody characterization in sera from responder and non-responder birds. **(A)** Opsonophagocytosis (OPA) assay. All sera (from Experiment A, B, and C) from negative control (PBS), responder (Res) and non-responder (N-Res) birds, collected on day 27 were pooled and evaluated for their ability to induce killing of *Campylobacter* by opsonophagocytosis. Each data point represents an independent replicate (each with fresh individually grown *Campylobacter* cells and individual blood samples from 35-day-old naïve broiler birds that served as a source for white blood cells). Each assay was carried out in triplicate, variations are shown by error bars. The bar represents the average for the four independent assays, error bars depict the standard error of the mean (SEM). Survival is expressed as percentage CFU compared to pooled sera from the negative control group (set to 100%). Statistically significant differences (two-tailed *t*-test, *p*<0.05) are indicated by an asterisk. **(B)** Distribution of N-glycans in total chicken serum analyzed by PGC LC ESI MS/MS. Individual N-glycans as identified from 10 individual responders and five non-responder birds have been summed according to groups representative for biosynthetic features (e.g., core-fucosylation=FUT8 activity, sialic acid linkage type, and structure class). **(C)** Distribution of N-glycans in enriched chicken IgY analyzed by PGC LC ESI MS/MS. Purified total IgYs from of four individual responders and five non-responder birds were analyzed. Statistical significance was calculated using two-way ANOVA wherein the value of *p* was ≤0.0001.

Pooled sera from all responder and non-responder birds identified in Experiments A, B, and C were tested for their ability to induce antigen-specific killing of *C. jejuni* 81-176 (Figure 3A). In comparison to bacteria incubated with pooled serum from the negative control groups (naïve chickens), an average of 21% bacterial killing was observed with pooled sera from non-responder birds, whereas an average of 54% bacterial killing was observed with pooled sera from responder birds. This result indicates that chicken antibodies targeting the *C. jejuni* N-glycan are indeed opsonizing, and further demonstrated that sera from responder birds display a significantly higher *Campylobacter*-killing activity when compared to sera from non-responder birds.

### Mass Spectrometric Analyses of Serum and Antibody Glycosylation Profiles

Different glycan profiles might result in different antibody avidities, i.e., in a stronger binding to the antigen and even though antibody levels could not be correlated with protection between responder and non-responders, variations in the glycosylation profile could increase antibody potency and could explain the observed differences in bacterial killing in the opsonophagocytosis assay. Therefore, we analyzed the total chicken serum and the IgY N-glycomes to evaluate potential differences in glycosylation between responders and non-responders by two orthogonal glycomics technologies (PGC nano LC-ESI MS/MS and MALDI-TOF MS).

#### The N-Glycome of Total Serum Glycoproteins and IgY in Responders and Non-responders

The chicken serum N-glycome was found to be comprised of at least 37 different N-glycan structures (Supplementary Figures S3A,B) with biantennary di-sialylated (α2-6/2-6 linked NeuAc) and biantennary mono-sialylated (α2-6 linked NeuAc) N-glycans contributing to approximately 85% of all N-glycans present. Next, we analyzed the N-glycome of purified IgY fractions by PGC nano LC-ESI MS/MS (Supplementary Figure S4) and found that chicken IgYs exhibited at least 27 different N-glycans that mirrored the major structures found for the total serum N-glycome.

#### Global Serum and IgY N-Glycan Profiles in Responders Versus Non-responders Remained Largely Unaltered

We then analyzed the distribution of N-glycans in total chicken sera by PGC LC-ESI MS/MS from 10 responders and five non-responders. We found that non-responders showed a slight (but not significant) tendency toward an increase of α2-3 linked NeuAc containing glycans, neutral, hybrid, and core fucosylated N-glycans (Figure 3B). In contrast, responders showed slightly (not significant) higher α2-3/2-6 linked NeuAc containing glycans, whereas no change was seen in the expression of α2-6 linked NeuAc containing glycans. For sialic acid-carrying N-glycans, responders showed a small, but significant increase in mono-sialylated glycans, while non-responders expressed a slightly significantly higher amount of di-sialylated glycans (Supplementary Figure S5A). A similar trend (albeit not significant) was observed for tri-sialylated glycans.

Analyses of individual core fucosylated glycans in total serum revealed that the amount of α2-6 di-sialylated biantennary core fucosylated glycans was significantly increased in non-responders (Supplementary Figure S6A). A similar trend was seen in α2-3 di-sialylated biantennary core fucosylated glycans and for one of the isomers of α2-6 mono-sialylated biantennary core fucosylated glycans, but the differences in abundance of the latter two were not statistically significant. Similarly, enriched IgY preparations from non-responders also revealed a trend toward an increase in the abundance of mono- and di-sialylated biantennary core fucosylated N-glycans; however, the observed differences were also not statistically significant (Supplementary Figure S6B).

Further analyses of the global serum N-glycans from a larger cohort of chickens (42 responders and 22 non-responders) by MALDI TOF Mass Spectrometry (MALDI-TOF MS) revealed a slight significant increase in the level of α2-6 linked NeuAc containing N-glycans in responders while non-responders showed a significant increase in α2-3/2-6 linked NeuAc containing N-glycans and a non-significant increasing trend in oligomannose, neutral, core fucosylated, and 2-3 linked NeuAc carrying N-glycans, respectively (Supplementary Figure S7). Further analysis of individual core fucosylated N-glycans revealed that although the abundance of α2-6 mono-sialylated biantennary core fucosylated glycans in total serum was significantly increased in non-responders, the distribution of other core fucosylated N-glycans remained unaltered or displayed very little change between the two groups (Supplementary Figure S8).

Similar to PGC LC ESI MS/MS (Supplementary Figure S5A), MALDI-TOF MS analysis of sialylated N-glycans in total chicken serum (Supplementary Figure S5B) showed a small (statistically significant) increase in mono-sialylated glycans in responders, while non-responders showed slightly significant higher levels of di-sialylated and tri-sialylated glycans. Chicken IgY N-glycans analyzed by PGC LC-ESI MS/MS (Figure 3C) showed that in the smaller cohort of responders and non-responders (four responders and five non-responders), the N-glycan profiles followed the same trend, a small, but not statistically significant increase in the expression of core fucosylated, oligomannose, and α2-3 and α2-3/2-6 linked NeuAc containing glycans that was found for total serum (see also Figures 3B,C, Supplementary Figures S5A,B, S6A,B, S7, S8).

### GWAS, Analysis of the Chicken Host Genetics: Responders Versus Non-responders

To investigate if the responder/non-responder effect is manifested through specific properties of the host itself, a GWAS of the response to vaccination was conducted. To identify single-nucleotide polymorphisms (SNPs) and genomic regions potentially associated with vaccine phenotypes, the GWAS was done using SNP data (*n*=475,058) from all vaccinated birds (*n*=134; 88 responders and 46 non-responders) and was analyzed following the SNP set approach described by Wu et al. (2010). Population stratification depicted by MDS plots indicate that for the top three dimensions, the 134 chickens can be divided into two sub-populations (Supplementary Figure S9A). However, no obvious association between the phenotype (responders or non-responders) and the population structure could be found (chi-square test; *p*=1). The results of PCA also showed that the top principal components accounted for only 2.5% of the total variation, suggesting that the population structure did not need to be integrated into the association analysis. Association analysis after FDR adjustment for multiple testing also showed no significant (FDR<0.1) association between SNPs and the responder versus non-responder phenotypes (Supplementary Figure S10). Further analysis revealed that the population structure resulted from the differences between the single experiments, as the smaller sub-population was enriched with chickens from Experiment A, while the larger sub-population was enriched with chickens from Experiments B and C (Supplementary Figure S9B). This could be due to a change in the flock generation and/or be due to a change in supplier of the birds. However, the exact reason for the observed differences could not be determined and GWAS data strongly suggest that the differences in vaccine responses are not due to genetic differences or variations in the chicken host.

In addition to the genotype, we also investigated if the gender of the birds might have an influence on colonization and vaccine responses. Analysis of 26,502 SNPs on the Z chromosome and 18 SNPs on the W chromosome from each bird did not reveal any evidence for an association between sex and the vaccine response phenotype [chi-square test (*p*=0.74)] (Supplementary Figure S11).

### Microbiota Analyses of Responder Versus Non-responder Birds

To analyze a potential role of the gut microbiota in the variable responses toward vaccination, we compared the cecal bacterial community between responder and non-responder birds. The composition of the cecal bacterial community was determined from each bird from the three vaccine experiments (total *n*=134; 88 responders and 46 non-responders) by Illumina sequencing of the V6-V8 region of the 16S rRNA gene. Considering that the composition of microbial communities could be affected by environmental, stochastic, and host -related factors, random forest analysis was performed using transformed OTU abundances to determine the out of bag (OOB) estimate of error rate for the main environmental and host variables that could potentially impact the microbial communities. These include experimental group (environmental factor), sex, and response (host factors). The OOB predicted error was estimated to be 1.5% if the analysis of the microbial data was done by experiment, but it increased to 13.53% if all samples were analyzed together by response (Supplementary Figure S12A). These results were supported by the goodness of fit test to determine significance of variables in unconstrained ordination (envfit function of the vegan package), which identified the “experiment” as the only significant variable impacting communities (*r*^2^=0.4702 implying moderate correlation, *p*<0.001; Supplementary Figure S12B). Clustering of all samples by PCA of Bray-Curtis dissimilarities also revealed clustering of samples by experiment (Supplementary Figure S12C) rather than by response (Supplementary Figure S12D). Overall, this analysis indicated that the samples from the three experiments should not be analyzed together as confounded by the experiment. The microbiota of birds from experiment A was vastly different to that of experiments B and C. Envfit analysis of abundance at the level of genera (Supplementary Figure S12B) showed that differences were especially dominant in the occurrence of *Bacteroides* spp., which were present at an average of 45.9% in experiment A, but with less than 0.02% on average in birds of experiments B and C.

PERMANOVA analysis on Bray-Curtis distances was performed for each individual experiment to determine differences in microbial community composition between responders and non-responders. Significant differences were only found for experiment A (Supplementary Figure S13A). Analysis of Bray-Curtis dissimilarities between individual birds as a measurement of β-diversity revealed a higher level of inter-individual variation between responder and non-responder birds for experiments A and C, but not for experiment B (Supplementary Figure S13B). Interestingly, within group inter-individual β-diversity is higher for responders in experiment A, but seems lower in experiment C, implying that these findings are not consistent and might be due to stochastic differences between experiments. Analysis of α-diversity showed statistically significant differences between responders and non-responders for birds in experiment C (*p*=0.0023). Noteworthy, there is a tendency for α-diversity to be lower in responders than in non-responders in all experiments (Supplementary Figure S13C).

**Figure 4:**
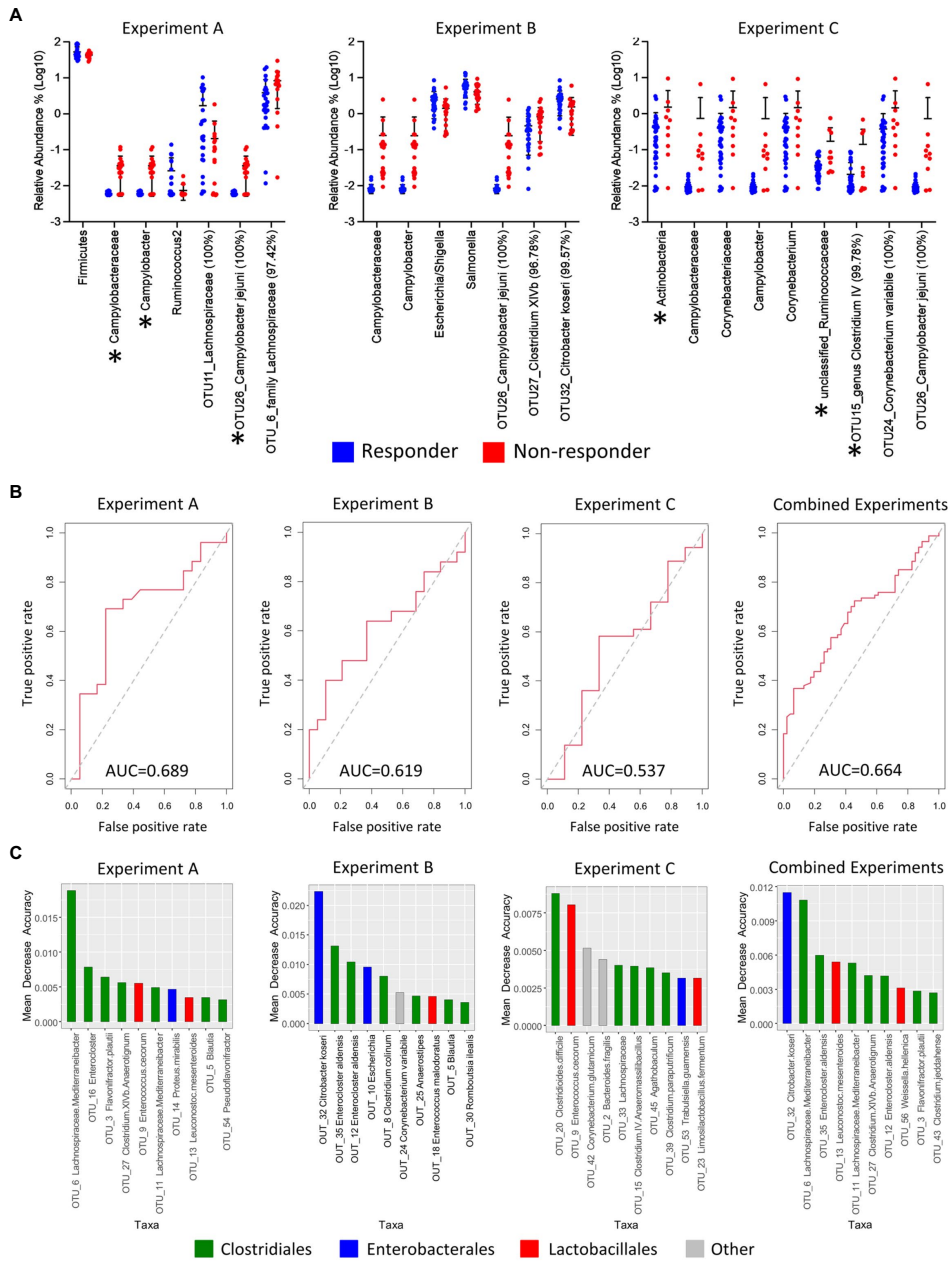
Significant taxonomic changes between responder and non-responder birds, receiver operating characteristics (ROC) plots, and identification of taxa that are predictive of response per experiment. **(A)** Scatter dot plot of statistically significant differences in taxa between responders and non-responders analyzed by multiple *t*-tests followed by false discovery rate (FDR) test using two-stage linear step-up procedure of Benjamini, Krieger, and Yekuteli. Lines represent mean and standard deviation. Percentages beside the names refer to percent identity for each taxon. Significance was established at *p*<0.05 and *q*<0.2. Asterisks indicate significant taxa after FDR. Lines represent mean and standard deviations. **(B)** ROC plots (using OTUs and excluding *Campylobacter* from the equation) were generated for the response factor and area under the curve (AUC) was calculated for individual experiments and for all experiments combined using the ROCR package in R statistical software. **(C)** Bar plots of variance importance per experiment as determined by mean decrease accuracy as part of random forest analyses. OTU, operational taxonomic unit; Un, unclassified.

Comparisons of cecal microbiota composition between responder and non-responder birds at different taxonomic levels showed significant differences. The clearest difference was detected for the family *Campylobacteraceae*, the genus *Campylobacter*, and an OTU assigned to *Campylobacter jejuni*, all of which were much lower in responders, confirming the effect of the vaccine in this subgroup of birds. Effects in other taxa were more subtle showing differences in genera within the order *Enterobacterales*, such as *Escherichia*/*Shigella*/*Salmonella*, in addition to differences observed in genera and species belonging, for example, to the Clostridium IV and XIVb groups within the order *Clostridiales* (Figure 4A). While *Enterobacterales* seem to be higher in responder than non-responder, the observed trend for *Clostridiales* is the opposite. Although we found significant differences among responder and non-responder, the findings were not consistent among all experiments, as it also has been observed in our previous work (Nothaft et al., 2017). However, there is some consistency in that *Clostridiales* or *Clostridiales*-related taxa (*Ruminococcus* and *Lachnospiraceae*) were again detected as being significantly different between responders and non-responders, also in agreement with findings from our previous studies (Nothaft et al., 2016, 2017).

In order to determine if the microbiota can predict the response to the vaccine, random forest analyses, using OTUs (Figure 4) and higher-level taxa (Supplementary Figure S14), were used to establish classifiers. *Campylobacter* was removed from the equation since the responder/non-responder groupings were determined by whether or not they were colonized with *Campylobacter*. Utility of models was examined using receiver operating characteristics (ROC) plots. The area under the curve serves to summarize the performance of the classifier into a single measure, with bigger values indicating improved accuracy of prediction. The area under the curves for experiments A, B, and C were estimated to be 0.639, 0.535, and 0.654 when using genera (Supplementary Figure S14A) and 0.689, 0.619, and 0.537 when using OTUs (Figure 4B). The random forest classifier for the combined data from all three experiments showed an area under the curve of 0.628 (using genera) and a slightly higher value of 0.664 when using OTUs. This finding indicated that there were major differences between experiments and that microbiota composition might not be a good predictor of the vaccine response. However, even though identified taxa had low predictive values, several OTUs that were significantly different between responders and non-responders (Figure 4A) were also identified to have a higher predictive value in the variable importance test (Figure 4C), and several fell within the same genera (e.g., *Enterocloster* and *Enterococcus*). In addition, genera within the *Clostridium* spp., *Ruminococcaceae* and *Lachnospiraceae* showed albeit low predictive potential (Supplementary Figure S14B), indicating that *Clostridiales* and *Clostridiales*-related taxa could be a variable in the response equation, supporting our previous findings (Nothaft et al., 2016, 2017).

### Cecal Contents Isolated From Responder Birds Improve Vaccine Efficacy

**Figure 5:**
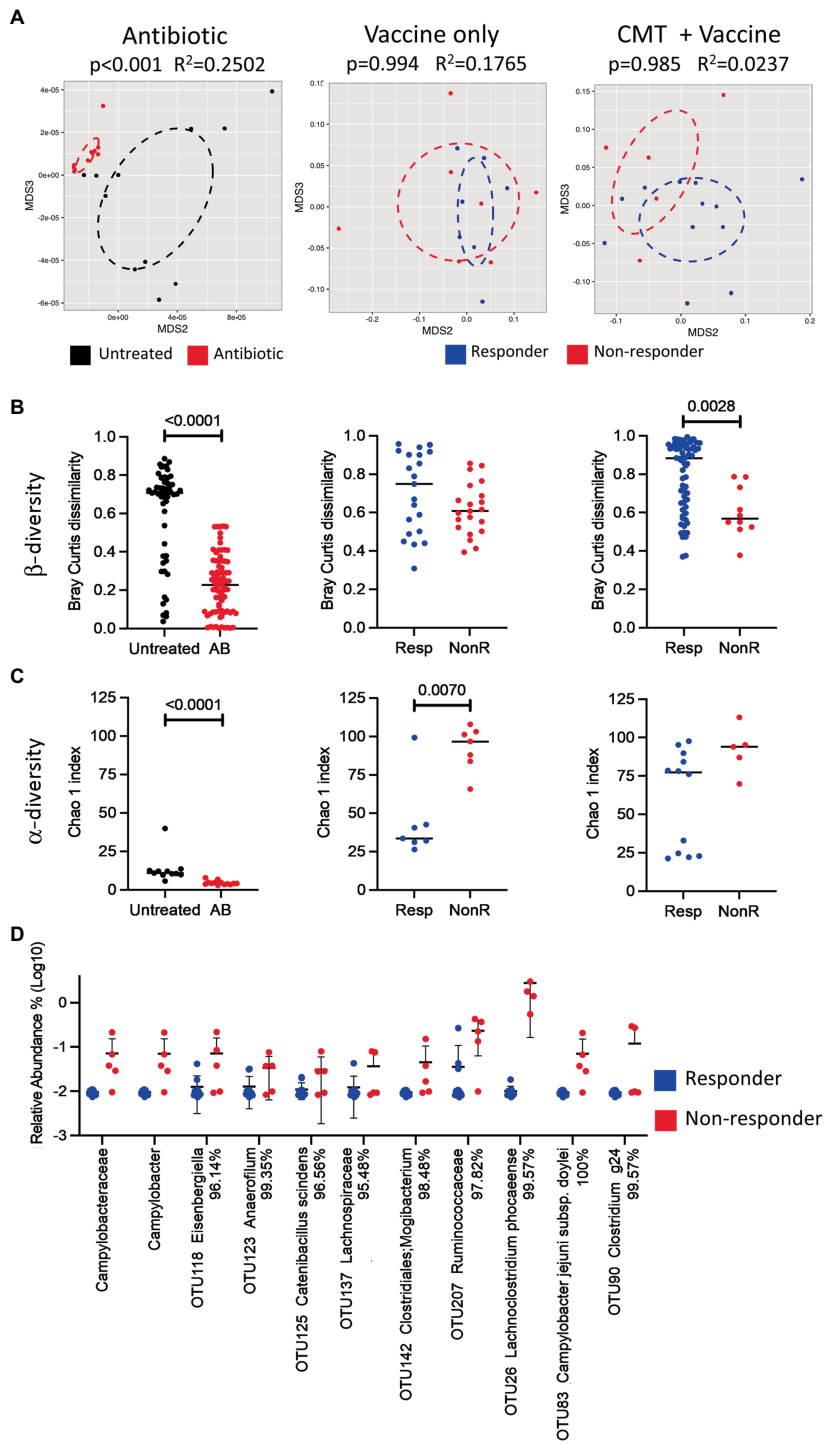
Effect of antibiotic treatment, vaccine, and cecal microbiota transplant on microbial diversity. **(A)** NMDS plots analyzed by PERMANOVA test; **(B)** scatter dot plots of Bray-Curtis dissimilarity analyzed by Mann-Whitney test as a measurement of beta diversity; and **(C)** scatter dot plots of Chao 1 indexes as a measure of alpha diversity for untreated and antibiotic-treated groups (left), and between responders and non-responders for vaccinated chickens treated with (right) or without (middle) cecal microbiota transplant. OTUs were used for all diversity analyses. Lines represent median values. **(D)** Scatter dot plot of statistically significant differences in taxa between responders and non-responders analyzed by multiple *t*-tests. Percentages beside the names refer to percent identity for each taxon. Lines represent mean and standard deviation. Significance was established at *p*<0.05. CMT, cecal microbiota transplant; AB, antibiotic; Resp, responder; and NonR, non-responder.

To determine the causal role of differences in the cecal microbiota in vaccine response, we conducted smaller experiments that explored the effect of cecal microbiota transplantation (CMT) on vaccine performance (*n*=9 for each study, *n*=18 combined). First, we investigated if using a cocktail of antibiotics reduces the native microbiota to subsequently increase engraftment. In an independent study, we then applied the same procedure to birds that received the cecal transplant. Subsequent analyses (by 16S rRNA sequencing) of the microbiota of birds that received the antibiotic cocktail resulted in a significant reduction of both inter-individual β-diversity (PERMANOVA of Bray-Curtis dissimilarities) and α-diversity (Chao 1 index), respectively (*p*<0.001 for each of the three analyses; Figures 5A–C, left columns), indicating that the antibiotic treatment was effective in reducing microbial diversity in the birds.

**Figure 6:**
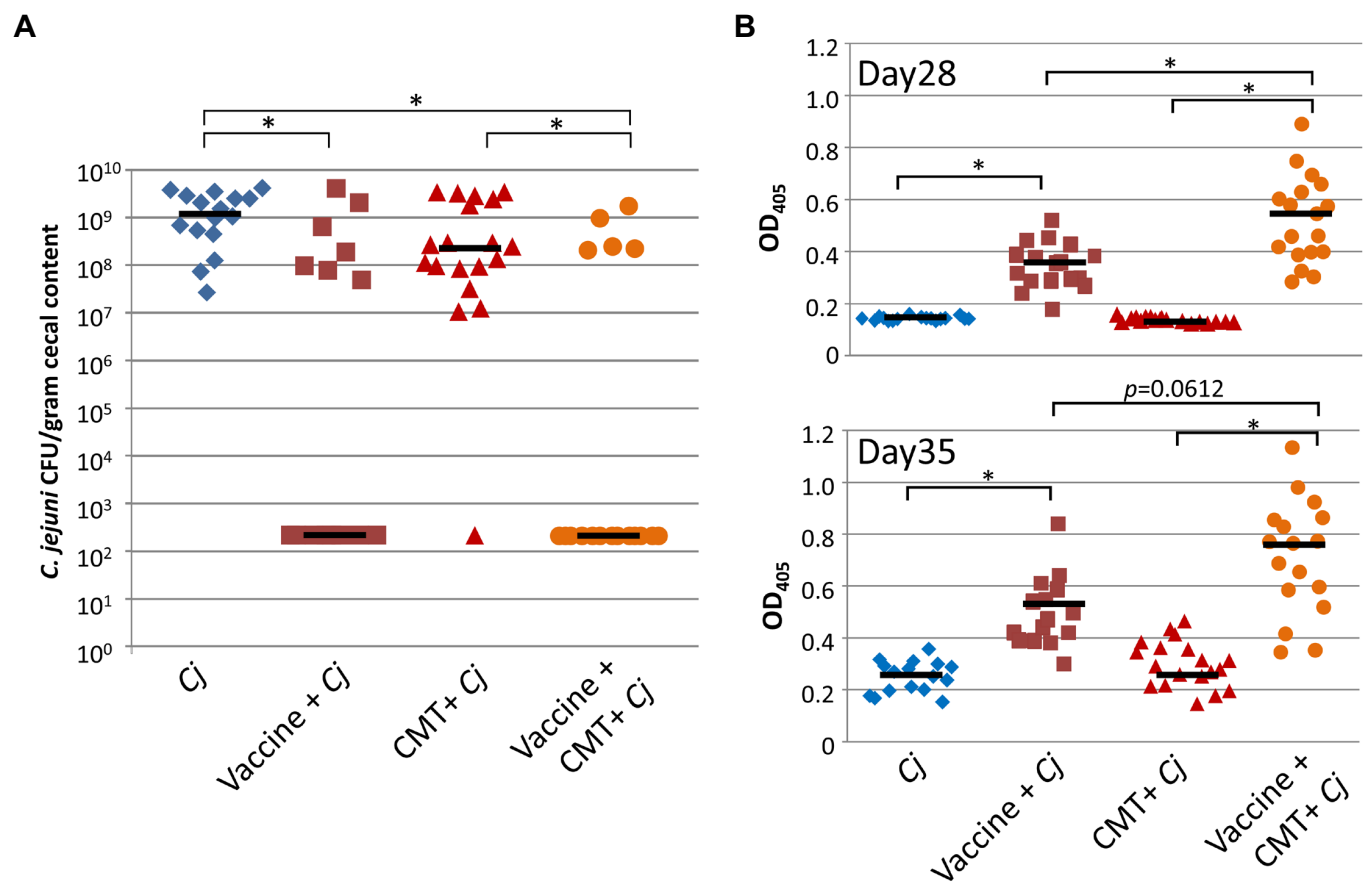
Cecal bacteria isolated from protected birds significantly improve vaccine performance. Combined results from two independent 35-day broiler vaccination studies. **(A)** Colonization of 35-day-old broiler birds*. C. jejuni* colonization [expressed as colony forming units (CFU) per gram of chicken cecal contents] after vaccination with 1×10^8^ live *E. coli* vaccine co-administered with cecal bacteria (Vaccine + CMT+*Cj*, *n*=18) compared to birds that received the live *E. coli* vaccine alone (Vaccine + *Cj*, *n*=15). To deplete the microbiota birds in the Vaccine + CMT+*Cj* as well in the cecal bacteria challenge control (CMT+*Cj*, *n*=19) groups, birds received antibiotics in the drinking water for 3days prior to vaccination/cecal microbiota transplantation. Birds in the positive control group (*Cj*, *n*=16) were not vaccinated but challenged with 1×10^6^ *C. jejuni* 81-178 on day 28. Statistically significant differences between groups (Fisher’s exact test, *p*<0.001) are indicated by an asterisk. **(B)** The N-glycan-specific immune responses. ELISA results using a 1:10 dilution of chicken sera from bleeds taken before challenge (day 28, upper panel) and at the day of euthanasia (day 35, lower panel). Each point represents the N-glycan-specific IgY antibody response measured at OD_450_ for each individual chicken. Black bars represent the median for each group. Statistically significant differences between groups (two-tailed *t*-test, *p*<0.05) are indicated by an asterisk.

We then compared efficacy of the APEC O:78-based N-glycan vaccine in birds that received the cecal microbiota isolated from responder birds versus birds that did not receive a CMT. In birds that received the CMT in combination with the vaccine, a lower percentage of *Campylobacter* colonization (after vaccination and challenge) was observed when compared to birds that received the vaccine alone (Figure 6A). In particular, we could demonstrate that the standard vaccination regimen on day 7 and day 21 with the vaccine alone resulted in 46.7% of birds still being colonized, whereas only 27.8% colonization was observed in birds that received the responder bird CMT in combination with the vaccine after antibiotic treatment (Figure 6A); however, this difference in colonization was not statistically significant (Fisher’s exact test, *p*=0.29). All birds in the positive control group (not vaccinated, but challenged) were found to be colonized and all except one bird in the group that received the CMT, but did not get vaccinated, was colonized with *Campylobacter* at similar levels. However, we observed statistically significant higher vaccine antigen-specific antibody titers (determined by two-tailed *t*-test) in the vaccine CMT group when compared to the vaccine alone group in serum prepared from blood taken before challenge (day 28, Figure 6B upper panel) and at the day of euthanasia, day 35 (Figure 6B, lower panel), suggesting that the microbiota of previously protected birds has a positive effect on the vaccine-induced immune response.

We further compared the microbial composition (by 16S sequencing) of the cecal contents at the trends suggesting that OTU classified as *Clostridium* spp., *Ruminococcaceae* and *Lachnospiraceae* are involved in the separation of both groups day of euthanasia (day 35) in responder and non-responder birds from the group that received the standard vaccination regimen (vaccine alone) as well as the group that received antibiotic treatment and CMT in combination with the vaccine. The results indicate that the alpha and beta diversities were different in responder versus non-responder birds (Figures 5A–C, middle and right columns, respectively) with (Figure 5D) consistent with what has been observed in the above-described experiments A, B, and C.

### Challenge With Persister *C. jejuni* Has no Effect on Vaccine Efficacy

**Figure 7:**
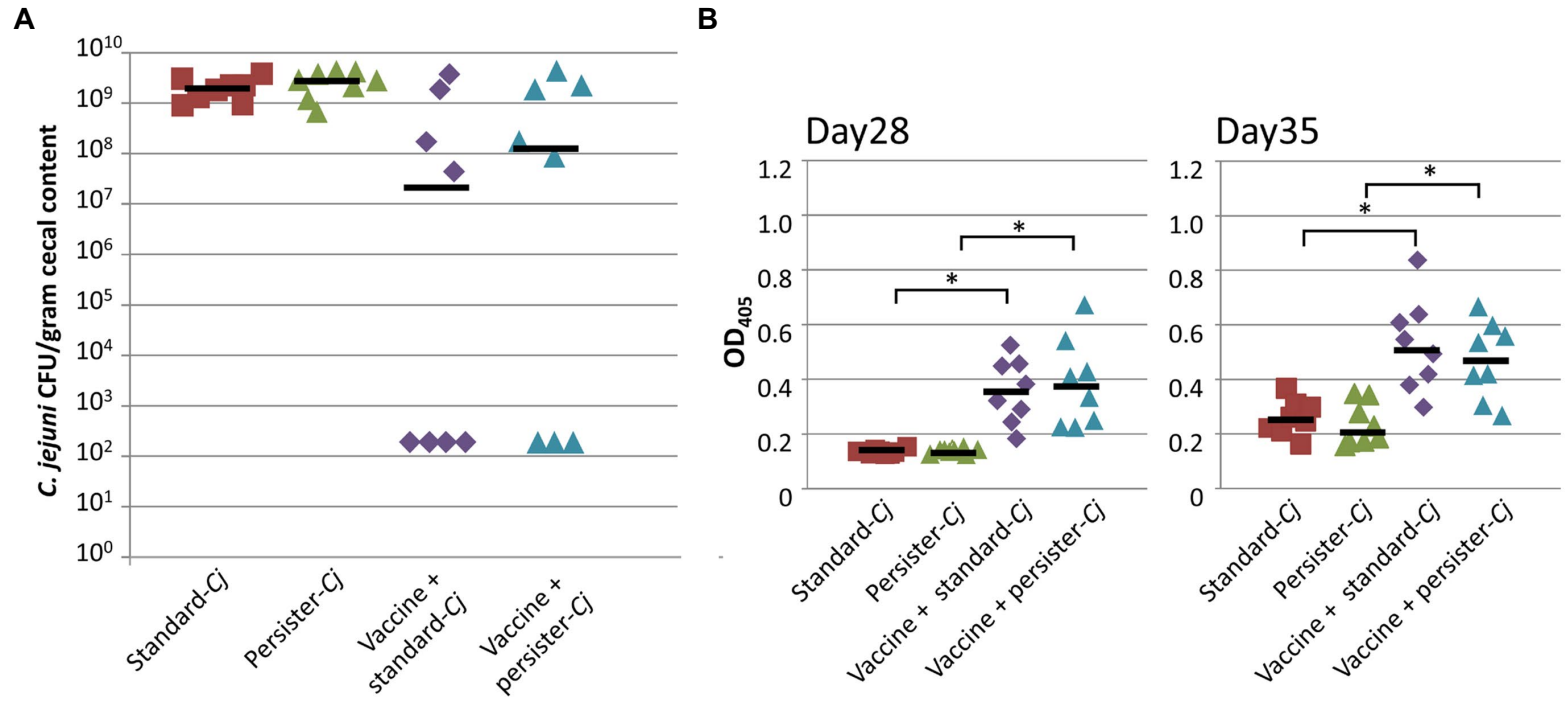
Challenge with “persister” *C. jejuni* 81-176 has no effect on vaccine performance. **(A)** Colonization of 35-day-old broiler birds. *C. jejuni* levels [expressed as colony forming units (CFU) per gram of chicken cecal contents] after vaccination (with 1×10^8^ live *E. coli* vaccine on day 7 and 21) and challenge. Birds were challenged with either 1×10^6^ “standard” *C. jejuni* 81-176 (standard-*Cj*, *n*=8) or with 1×10^6^ *C. jejuni* 81-176 recovered from vaccinated bird that were still colonized (persister-*Cj*, *n*=8) on day 28. Standard-*Cj* and persister-*Cj* groups (*n*=8) were not vaccinated, but challenged on day 28. No statistical difference in CFU (Fisher’s exact test) was observed between the standard and persister groups. **(B)** The N-glycan-specific immune responses. ELISA results using a 1:10 dilution of chicken sera from bleeds taken before challenge (day 28, upper panel) and at the day of euthanasia (day 35, lower panel). Each point represents the N-glycan-specific IgY antibody response measured at OD_450_ for each individual chicken. Black bars represent the median for each group. ^*^statistically significant; two-tailed *t*-test, *p*<0.05.

Our previous studies did not detect any changes in phase-variable gene expression in our challenge strain, *C. jejuni* 81-176 (Nothaft et al., 2016, 2017; Wanford et al., 2018) capable of persisting in non-responder birds; however, the limited analyses did not test for other SNPs in the genome of *Cj*-81-176 that could potentially result in the evolution of a *C. jejuni* super-colonizer or persister strain. We therefore collected *C. jejuni* isolates from non-responder birds and used those pooled isolates in a subsequent challenge study. Similar to Experiments A, B, and C, we observed a bimodal distribution in colonization after a second dose vaccination and challenge regimen. Challenge with the “standard” *C. jejuni* 81-176 (group three, *n*=8) or the “persister” *C. jejuni* pool (group four, *n*=8) did not result in statistical differences in protection. All birds in both positive control groups (not vaccinated, but challenged with either the standard or the persister pool of *C. jejuni*) were colonized at similar levels (Figure 7A). In addition, comparable levels of N-glycan-specific IgYs (determined by ELISA) were be found in the sera of vaccinated birds from groups three and four isolated on day 28 and day 35 (Figure 7B).

## Discussion

Oral administration of an attenuated *E. coli* strain engineered to express the *C. jejuni* N-glycan reduces bacterial colonization in leghorns and broilers, and thus has great potential to prevent pathogen entry into the food chain. However, previous studies showed that a fraction of birds was still colonized after vaccination (Nothaft et al., 2016, 2017), and this was recapitulated in Experiments A, B, and C described here. Genome-wide chicken genotyping revealed no statistically significant genetic difference between responder and non-responder birds, indicating the differential susceptibility to colonization or differences in vaccine responses are not associated with genetic traits. That contrasts with other studies that have shown that resistance to pathogens can be, at least partly, genetically determined. Differences in resistance to APEC among inbred chicken lines were reported, but were dependent on the infectious dose (Alber et al., 2019). Resistance loci to systemic salmonellosis were mapped (Mariani et al., 2001; Wigley et al., 2002, 2006; Fife et al., 2009) and refined to an 8kb, 14 gene region on chromosome 5, termed SAL1, including the CD27-binding protein and AKT1, a REAC-alpha serine/threonine protein kinase homolog, both involved in innate immune response signaling (Kaiser et al., 2009). For *Campylobacter*, over 150 genes potentially involved in natural resistance have been identified including (among other factors) genes related to the MHC complex and the innate and adaptive immune responses, cadherins and genes modulating cadherin functions, and genes for renin-angiotensin signaling (Kaiser, 2010; Connell et al., 2013; Psifidi et al., 2016, 2021). Although most of the identified loci were different to the ones identified for *Salmonella* resistance (Kaiser, 2010), increased macrophage functionality [e.g., the natural resistance-associated macrophage protein 1 (*NRAMP1*)] gene and increased expression of pro-inflammatory cytokines and chemokines have been associated with resistance to both pathogens (Boyd et al., 2005; Wigley et al., 2006; Psifidi et al., 2016); however, these associations were not observed here.

### Immune Responses and Antibody Properties as Potential Factors for Vaccine Efficacy

Similar immune responses to vaccination were induced in all our studies and the N-glycan-specific serum and cecal content supernatant IgY levels clearly distinguished vaccinated versus unvaccinated birds with increased antigen responses in the former compared to *C. jejuni* infection alone. In addition, in day 35 cecal samples, N-glycan-specific IgA could be detected at low levels, but there was no significant difference between challenged only and vaccinated birds which could be due to the shorter half-life of IgA versus IgY (Davis et al., 2020). Therefore, IgA may still contribute, but since samples were collected 7days after challenge (14days after the vaccine boost) our assay might not be fully representative for IgA levels present at the day of challenge. Intestinal IgY (in addition to IgA) has also been observed in other vaccination studies (Kulkarni et al., 2010; Annamalai et al., 2013; Wilde et al., 2019) and has been suggested to contribute to the reduction of the pathogen in the gut. However, the intestinal response to our oral *E. coli*-based vaccine would not explain the responder/non-responder effect (similar to the serum IgY response) but could in combination with other/unknown factors contribute to the observed reduction of *Campylobacter* in responder birds; similar to other studies that have shown that vaccine response does not necessarily correlate with protection (Chintoan-Uta et al., 2015). In general, B-cell responses to *C. jejuni* colonization in chickens show that IgY levels correlate with the presence of the pathogen in the intestine, but natural antibody levels in the cecum are not sufficient to clear the bacteria (Lacharme-Lora et al., 2017). However, it has also been shown that IgY antibodies are protective in the first 2weeks of life and that this effect can be prolonged by oral administration of pathogen-specific IgY (Vandeputte et al., 2019). We also demonstrated that antisera from responder birds showed a higher *Campylobacter*-killing activity, indicating that sera from those birds might possess a higher affinity for the antigen or the antibody pool is enriched with IgYs with desired effector functions. It has been shown that human antibody N-glycosylation (IgG glycan at N297) and the structure of the N-glycan are important for antibody functionality (Krapp et al., 2003; Jefferis, 2009). For example, changes in IgG glycosylation play a role in autoimmune disease and cancer in humans and impact viral and bacterial colonization (Chen et al., 2020; Irvine and Alter, 2020). But little is known about IgY glycosylation and its contribution to antibody functionality in chickens. Despite the differences in killing activities, we found that glycosylation (at least in serum) is very similar in responders and non-responders, although whole IgY preparations showed a trend, albeit insignificant, toward reduced fucosylation in responders. This indicates that vaccination does not change the global N-glycosylation profile. Although one might speculate that N-glycosylation profiles of antigen-specific IgYs could be different, anti-*C. jejuni* (N-glycan)-specific IgY constitutes only a minor subfraction of the total IgY and changes (if present) would not result in a significant difference in the global N-glycosylation profile. It has been shown that effector function of staphylococcal protein A antibodies in mice is indeed glycosylation-dependent. Here, galactosylation favors recruitment of the complement component C1q and is indispensable for killing of staphylococci (Chen et al., 2020). Similarly, afucosylated Abs protected against infection through enhanced Fcγ receptor engagement and phagocytosis (Chen et al., 2020). While a glycosylation-dependent impact on IgG function is undisputed in mammals, the functional role IgY glycosylation has in chickens is still unclear. However, from a producer/consumer point of view, the fact that vaccination does not induce any detectable impact on the qualitative and quantitative composition of the serum and total IgY N-glycome, our *E. coli* vaccine does not induce any health risk by generating potentially immunogenic glyco-epitopes that could be harmful for humans.

### The Chicken Microbiota and Its Effect on Vaccination

In general, the chicken gut microbiota is part of a complex ecosystem influenced by many factors, such as stocking density, stress, feed type, and other additives, that can either increase or decrease microbial diversity (Ijaz et al., 2018; Hankel et al., 2019; Kridtayopas et al., 2019). The composition of the microbiota has been reported to affect the ability of *Campylobacter* to reside in the chicken cecum (Han et al., 2017; Sakaridis et al., 2018; Ocejo et al., 2019). Increased diversity and increased environmental pressure on the microbial community favor the presence of *Campylobacter* (McKenna et al., 2020); however, feed additives did not change the alpha diversity of the cecal microbiota but decreased the *C. jejuni* cecal counts by 0.7 logs and were associated with an increase of *Bifidobacterium* species (Thibodeau et al., 2015). Studies in humans and mice indicate that the microbiome can also influence vaccine-induced immune responses (Jamieson, 2015; De Jong et al., 2020), but very limited information is available for other species. In our studies, OTU classified as *Clostridium* spp., *Ruminococcaceae* and *Lachnospiraceae* seem to be involved in the responder/non-responder effect after vaccination and challenge. Specifically, *Clostridiales*, but also other bacteria (Zhang et al., 2007), present in the natural microbiota contribute to the control of *C. jejuni* colonization, an effect postulated to occur by exclusion or immune-priming (Han et al., 2017). Interestingly, the *Clostridiales* effect is not restricted to the chicken model. *Clostridiales* and also higher relative abundances of *Lachnospiraceae* seem to have protective properties against *Campylobacter* infections in humans (Dicksved et al., 2014; Kampmann et al., 2016), and members of *Lachnospiraceae* were also enriched in our responder birds. In mice, the presence/enrichment of *Clostridium* cluster XI, but also of *Enterococcus faecalis* (Gram-positive lactic acid bacteria, Firmicute), *Bifidobacterium*, and *Lactobacillus* have been correlated with reduced *Campylobacter* infections (O’Loughlin et al., 2015; Sun et al., 2018), whereas antibiotic treatment (removal of those species) increases severity of colitis (Sun et al., 2018). On the other hand, numbers of enterobacteria were reported to potentially facilitate *C. jejuni* infection (Haag et al., 2012). Although the latter observation was made in infant mice, an effect of these bacteria in the chicken model cannot be ruled out since members of *Enterobacterales* were also affected in our studies.

Competitive exclusion of *Campylobacter* by potentially beneficial bacteria present in the cecal transplant obtained from responder birds can most likely be eliminated since the fecal transplant alone had no effect on *Campylobacter* colonization. Interestingly, it has been shown that the transfer of microbiota from a line of birds with a higher resistance to *Campylobacter* cannot exert the same protective effect in another line of birds that is naturally more susceptible to colonization. In fact, the transfer of the microbiota from the more resistant line further increased *Campylobacter* susceptibility of the less resistant line (Chintoan-Uta et al., 2020). However, the authors also reported that, among others, three specific OTUs from the *Ruminococcaceae* family differed between susceptible and more resistant lines (Chintoan-Uta et al., 2020). In line with this, we found members of the *Ruminococcaceae* family as a potential factor in distinguishing responder and non-responders. Another study showed that the introduction of the *Clostridiales*-dominated microbiota from eight-week-old birds into newly hatched chicks had a protective effect against *Campylobacter* colonization and altered the gut microbiota. However, no effect was observed when 7-day-old chicks received the transplant (Gilroy et al., 2018). This is in accordance with the “window of opportunity” for colonization that has been described in mice and suggested for human infants for the protective/immune stimulating effect of some bacteria (Torow and Hornef, 2017).

Moreover, *Campylobacter* infection was associated with decreases in the abundance of *Clostridium* cluster XIVa and *Lactobacillaceae* OTUs and also with an age-dependent shift in OTUs of members of the *Lachnospiraceae* and *Ruminococcaceae* families (Connerton et al., 2018). Based on the observation that transplanted bacteria only exist for a limited time (Chintoan-Uta et al., 2020), one might speculate that our live vaccine potentially creates a more favorable environment for the more beneficial bacteria to thrive in certain (responder) birds resulting in the complete elimination of *Campylobacter* or that *Campylobacter* changes the niche environment in the gut in its favor [a strategy described for other pathogens (Baumler and Sperandio, 2016)] and that the vaccine removes or reduces *Campylobacter*, which then redresses some of the shifts.

### In Summary

This study indicated that microbial composition and antibody function, but not host genetics or the serum glycome, may contribute toward the efficacy of the glycoconjugate vaccine in chickens. Although the gut microbiota has a low predictive value, this is likely due to the substantial variations between experiments that are not linked to the vaccine. However, certain OTUs that fell within the same genera (*Enterocloster* and *Enterococcus*) and within the *Clostridium* spp., *Ruminococcaceae* and *Lachnospiraceae* were still associated with responders and non-responders suggesting there may be functional redundancy among microbiotas as different taxa appear in different experiments. There are likely stochastic factors that influence the assembly of the microbial community resulting in inter-individual differences and subsequently in changes in the levels of certain microbial components that could function as adjuvants or impact immune responses.

Despite best efforts to hold environmental factors constant, a change in supplier (through the same distributer), time of the year during which the study, was conducted influencing temperature levels during transport, or even differences in animal handling during vaccination/challenge, as well as other minimal variations (e.g., changes in feed intake), were beyond the control of the study design. This could result in an overall different microbial composition not related to the vaccine, and that most likely generates noise. Such variations in the composition of the microbiota even between experimental repeats has also been observed in previous studies (Stanley et al., 2013) and could further influence vaccine efficacy and *Campylobacter* colonization potential and could make it difficult to increase vaccine efficiency. Our experiments exemplify the challenges experienced in many other areas of microbiome research. Microbiomes are clearly causal to many host phenotypes, but the identification of causal components is confounded by the high level of unexplained variation (Velazquez et al., 2019).

Future efforts could include whole microbiome metagenomics and metabolomics in combination with *ex vivo* immune assays to determine which fraction/compound/metabolite of the microbiota could impact immune cell functions in relation to vaccine activity. In addition, data could be collected at different time points of the study (especially prior to challenge) since having sequence data only at the end of the experiment limits correlations to the microbiota identified after the *Campylobacter* challenge and the effect of *Campylobacter* or the immune response in response to vaccination might alter the microbiome differently. However, our *E. coli* lipid A-core N-glycan expression system has been repeatedly shown to reduce *C. jejuni* CFU after vaccination and challenge, while other carriers do not provide this protective effect (Vohra et al., 2020). Therefore, optimization of the antigen carrier in combination with microbiome manipulations will potentially result in further improvement of vaccine efficacy and help to completely eliminate *Campylobacter* from the food chain.

## Data Availability Statement

All data generated as part of this study are included in the article and supplement. Source data file is available from the corresponding author. All raw MS data have been uploaded into the Glycopost repository and are available at the following link: https://glycopost.glycosmos.org/preview/135905020760cbc9cc23ffb; PIN CODE: 7191. The 16S rRNA datasets generated and analyzed during these studies are available in the NCBI SRA repository, accession numbers SAMN19776168–SAMN19776360 under BioProject PRJNA739190. The chicken GWAS data are available at GEO, accession number GSE181619.

## Ethics Statement

The animal studies and experimental procedures were carried out in accordance with the protocol (AUP00000003) reviewed and approved by the Animal Care and Use Committee at the University of Alberta.

## Author Contributions

HN, GP, JW, MM, and CS designed the experiments. HN and CS organized and executed the animal studies and all the immunological studies, and wrote the initial draft of the manuscript. HN constructed and verified the vaccine strain. AM and DK performed all mass spectrometry and analyzed the data. MM, TY, and GP performed the DNA extractions, 16S sequencing, and GWAS data analysis. MP-M and JW analyzed and interpreted all the 16S sequencing/microbiota data. All authors read, edited, and approved the final manuscript.

## Funding

Parts of this work were supported by the Alberta Livestock and Meat Agency (to HN, MM, GP, TY, MP-M, JW, and CS). CS was an Alberta Innovates Strategic Chair in Bacterial Glycomics. DK is the recipient of an Australian Research Council Future Fellowship (project number FT160100344) funded by the Australian Government. The glycomics work was made possible by a Griffith University Postgraduate Research Scholarship (GUPRS) awarded to AM.

## Conflict of Interest

HN is employed by VaxAlta Inc., and CS is affiliated with VaxAlta. MM is employed by Neogen. TY is currently employed by ST Genetics, but was not affiliated with the company at the time of the work.

The remaining authors declare that the research was conducted in the absence of any commercial or financial relationships that could be construed as a potential conflict of interest.

## Publisher’s Note

All claims expressed in this article are solely those of the authors and do not necessarily represent those of their affiliated organizations, or those of the publisher, the editors and the reviewers. Any product that may be evaluated in this article, or claim that may be made by its manufacturer, is not guaranteed or endorsed by the publisher.

## Footnotes

^1^ http://www.cdc.gov/nczved/divisions/dfbmd/diseases/campylobacter/

^2^ http://hannonlab.cshl.edu/fastx_toolkit/

^3^ www.graphpad.com

^4^ https://web.expasy.org/glycomod/

## Nomenclature

APEC: Avian pathogenic Escherichia coli
CFU: Colony forming units
Cj: Campylobacter jejuni
HCCA: alpha-cyano-4-hydroxycinnamic acid
HILIC: Hydrophilic interaction chromatography
MALDI-TOF MS: Matrix-assisted laser desorption ionization time-of-flight mass spectrometry
MOI: Multiplicity of infection
MS: Mass spectrometry
OTU: Operational taxonomic unit
PBS: Phosphate-buffered saline
PGC LC ESI MS/MS: Porous graphitized carbon liquid chromatography with electrospray ionization mass spectrometry
SNP: Single-nucleotide polymorphism
TFA: Trifluoroacetic acid
