# Supplementary material for: Improving Chicken Responses to Glycoconjugate Vaccination Against Campylobacter jejuni
Source: Front Microbiol. 2021 Nov 16;12:734526. doi: 10.3389/fmicb.2021.734526 (PMC8637857; doi:10.3389/fmicb.2021.734526)
Supplement: Supplementary file 1 [file Data_Sheet_1.pdf]

Supplementary Material

Results

Our previous studies have used an *E. coli* K12-derivative that expresses the protein glycosylation locus from a plasmid to generate a live vaccine strain that displays the N-glycan on its surface (Nothaft et al., 2016; Nothaft et al., 2017). Here, we designed a novel carrier strain for the N-glycan vaccine, an attenuated avian pathogenic *Escherichia coli* (APEC) serotype O:78(*aroA*) mutant that has the minimal biosynthetic N-glycosylation machinery of *C. jejuni* (*Cj*) stably integrated into the chromosome (Supplementary Figure S1A and S1B). For better traceability, the strain was further transformed with plasmid pACYC184 that confers chloramphenicol resistance. Western blot analyses with the *Cj*-N-glycan-specific antiserum, R1, confirmed expression of the *Cj*-N-glycan lipid A-core N-glycan fusion in the APEC O:78-based vaccine strain (O:78 *aroA wzy*::*pgl*::*kan* (pACYC184)) (Supplementary Figure S1C). A signal corresponding to the vaccine antigen migrated at a similar molecular weight when compared to the previously described *Cj*-N-glycan-lipid A produced in *E. coli* K12.

Figure S1A

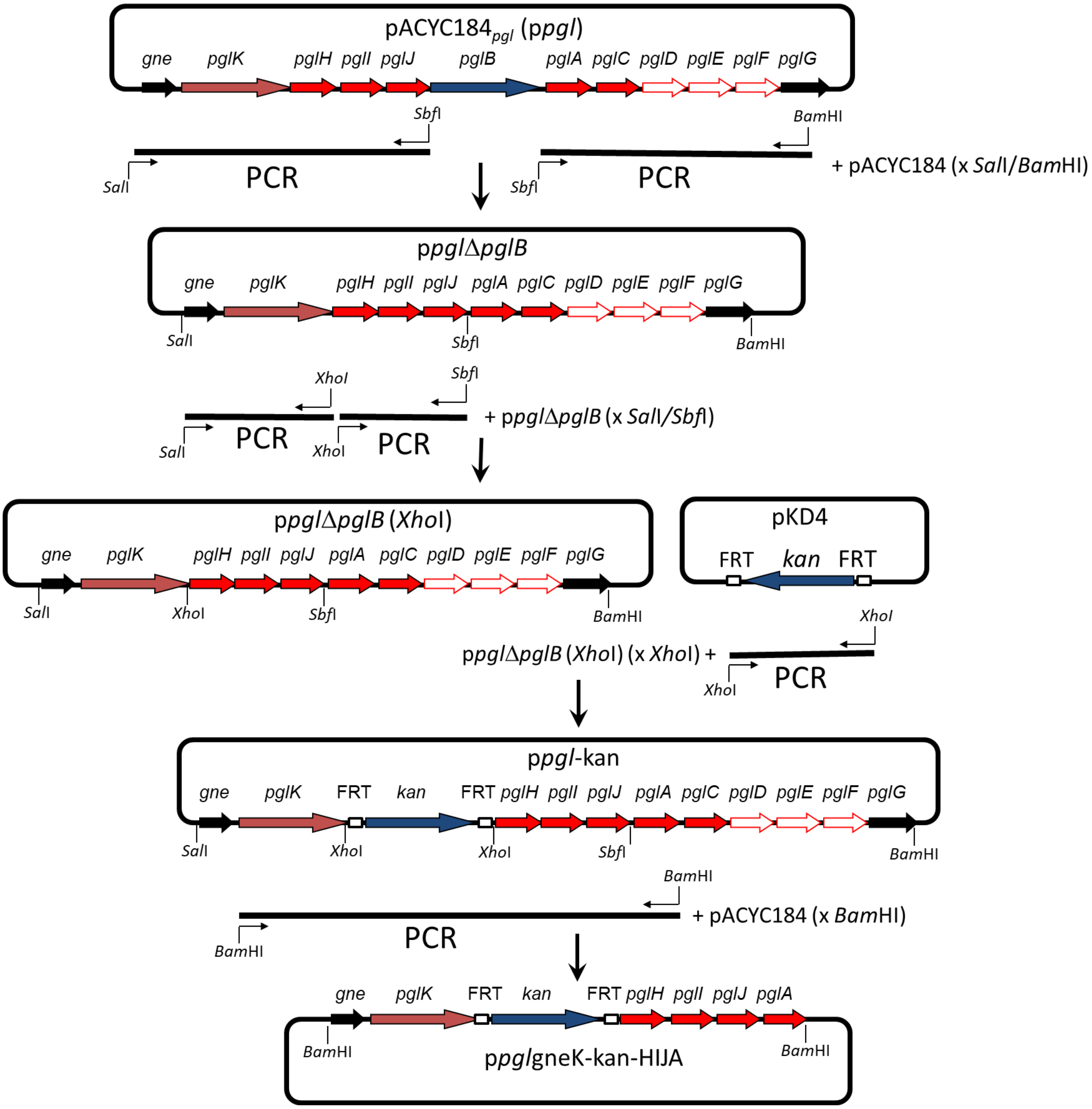
pACYC184pgl (ppgl)
gne pglK pglH pglI pglJ pglB pglA pglC pglD pglE pglF pglG
SbfI
BamHI
SalI
PCR
SbfI
PCR
+ pACYC184 (x SalI/BamHI)
ppglΔpglB
gne pglK pglH pglI pglJ pglA pglC pglD pglE pglF pglG
SalI
SbfI
BamHI
XhoI
SbfI
SalI
PCR
XhoI
PCR
+ ppglΔpglB (x SalI/SbfI)
ppglΔpglB (XhoI)
gne pglK pglH pglI pglJ pglA pglC pglD pglE pglF pglG
SalI
XhoI
SbfI
BamHI
pKD4
FRT kan FRT
XhoI
ppglΔpglB (XhoI) (x XhoI) +
XhoI
PCR
ppgl-kan
gne pglK FRT kan FRT pglH pglI pglJ pglA pglC pglD pglE pglF pglG
SalI
XhoI
XhoI
SbfI
BamHI
BamHI
+ pACYC184 (x BamHI)
BamHI
PCR
gne pglK FRT kan FRT pglH pglI pglJ pglA
BamHI
ppglgneK-kan-HIJA
BamHI

Figure S1B

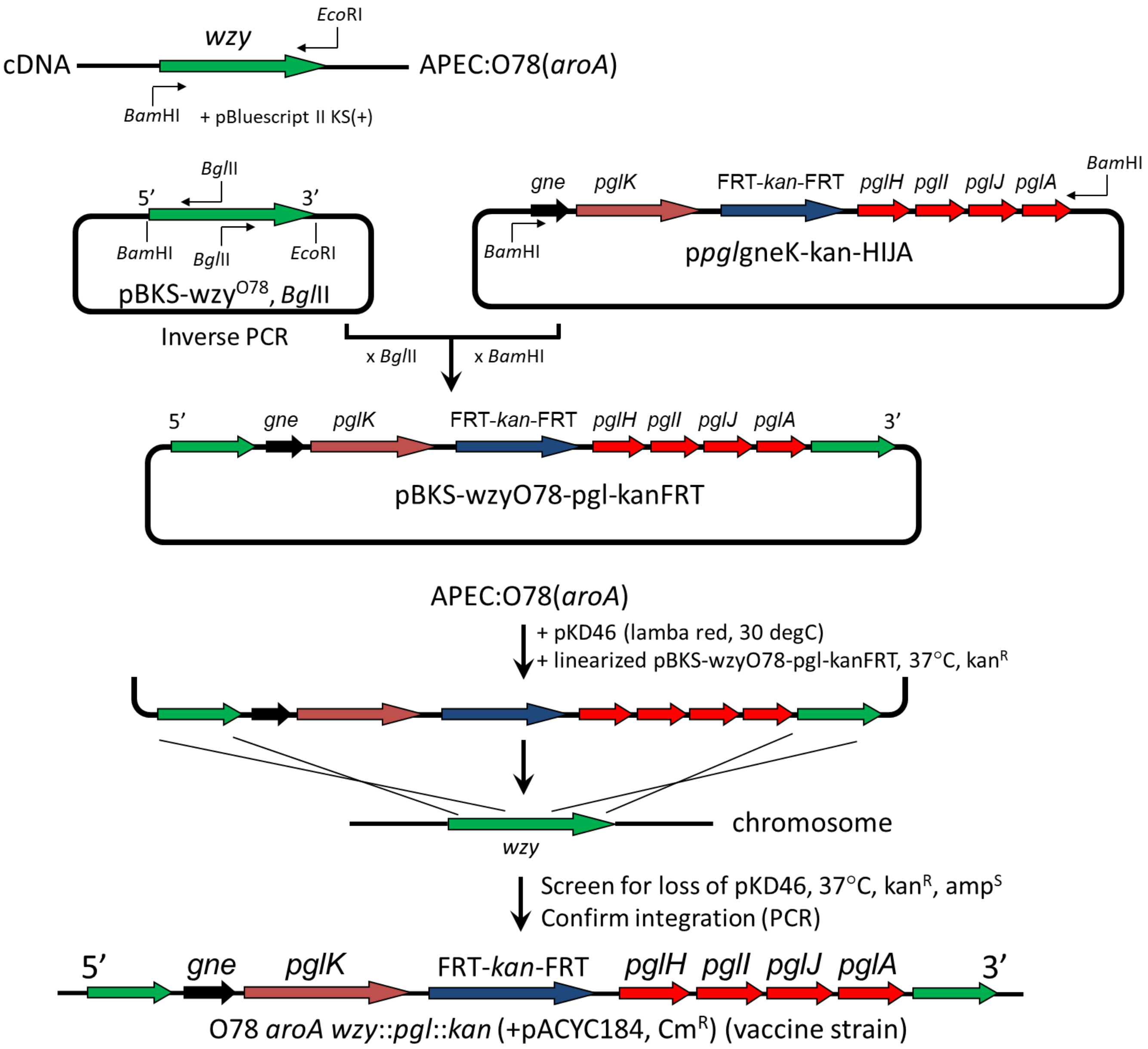
EcoRI
wzy
cDNA
APEC:O78(aroA)
BamHI
+ pBluescript II KS(+)
BglII
5'
3'
BamHI
BglII
EcoRI
pBKS-wzyO78, BglII
Inverse PCR
gne
pglK
FRT-kan-FRT
pglH
pglI
pglJ
pglA
BamHI
BamHI
ppglgneK-kan-HIJA
x BglII
x BamHI
5'
gne
pglK
FRT-kan-FRT
pglH
pglI
pglJ
pglA
3'
pBKS-wzyO78-pgl-kanFRT
APEC:O78(aroA)
+ pKD46 (lamba red, 30 degC)
+ linearized pBKS-wzyO78-pgl-kanFRT, 37°C, kanR
chromosome
wzy
Screen for loss of pKD46, 37°C, kanR, ampS
Confirm integration (PCR)
5'
gne
pglK
FRT-kan-FRT
pglH
pglI
pglJ
pglA
3'
O78 aroA wzy::pgl::kan (+pACYC184, CmR) (vaccine strain)

Figure S1C

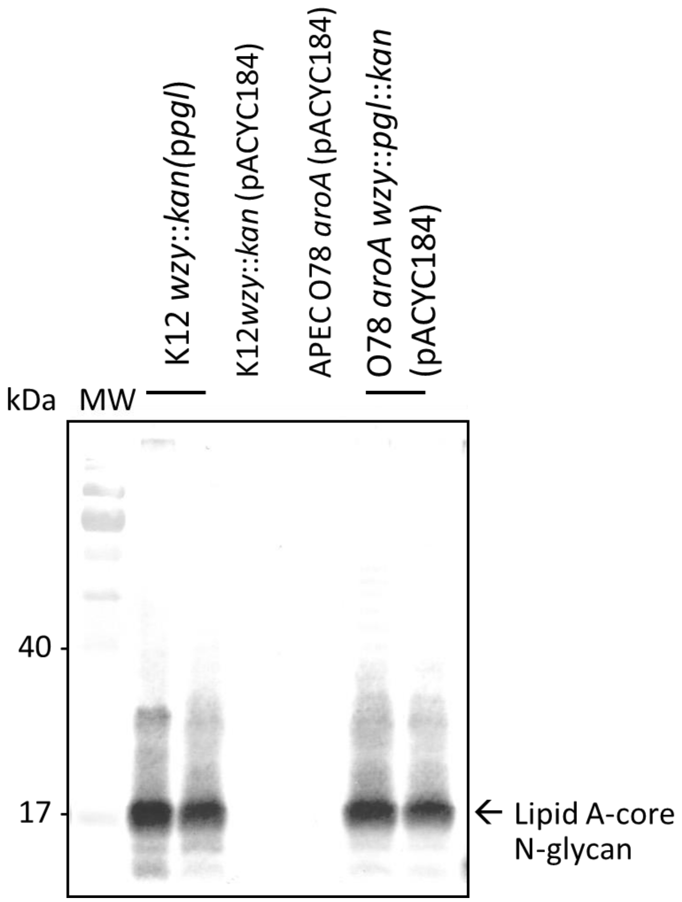

Figure S1. Construction and verification of the APEC O:78-based vaccine strain. Illustration depicting **(A)** the construction of the plasmid containing the minimal *pgl* operon genes and **(B)** construction of the plasmid for the integration of the minimal *pgl* operon genes into the *wzy* locus of APEC O:78(*aroA*). A detailed description of the procedure is provided in the manuscript. *C. jejuni pgl* operon genes (*pglH*, *pglI*, *pglJ*, *pglA* and *pglC*) encoding GTases are in red, genes encoding the flippase (PglK) and the oligosaccharyltransferase (PglB) are in brown and blue, respectively. Genes required for diNAcBac biosynthesis (*pdlD*, *pglE* and *pglF*) are depicted as open arrows, genes encoding Gne (an UDP-GlcNAc/Glc 4-epimerase) and PglG (no known function in N-glycan biosynthesis) are in black. **(C) Expression of the lipid A-core N-glycan compound in APEC O:78(*aroA*).** Western blot of proteinase K digested whole cell lysates of *E. coli* K12 *wzy*::*kan* (p*pgl*), *E. coli K12 wzy*::*kan* (pACYC184), APEC O:78 *aroA* (pACYC184) and O:78 *aroA wzy*::*pgl*::*kan* (pACYC184) probed with N-glycan-specific antiserum (R1) is shown. The formation of the lipid A-core N-glycan molecule is indicated by an arrow. Relevant molecular weight markers (MW in kDa) are indicated on the left.

Figure S2

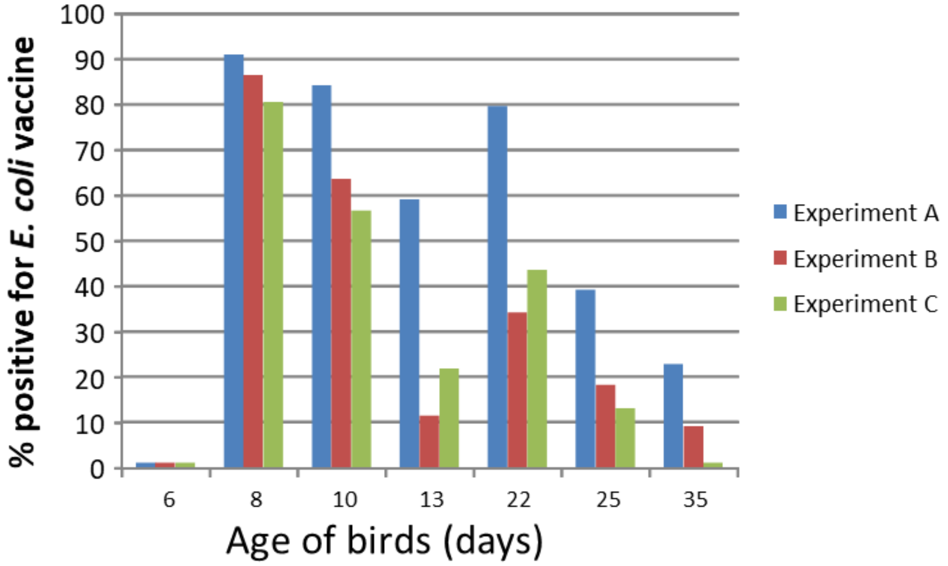

Figure S2. *E. coli* vaccine persistence. The *E. coli* APEC O:78 vaccine fecal shedding was inspected by cloacal swabs taken on the indicated days of the individual experiments. Vaccine persistence is expressed as percent of positive birds within each group with detectable colonies on selective plates.

Figure S3

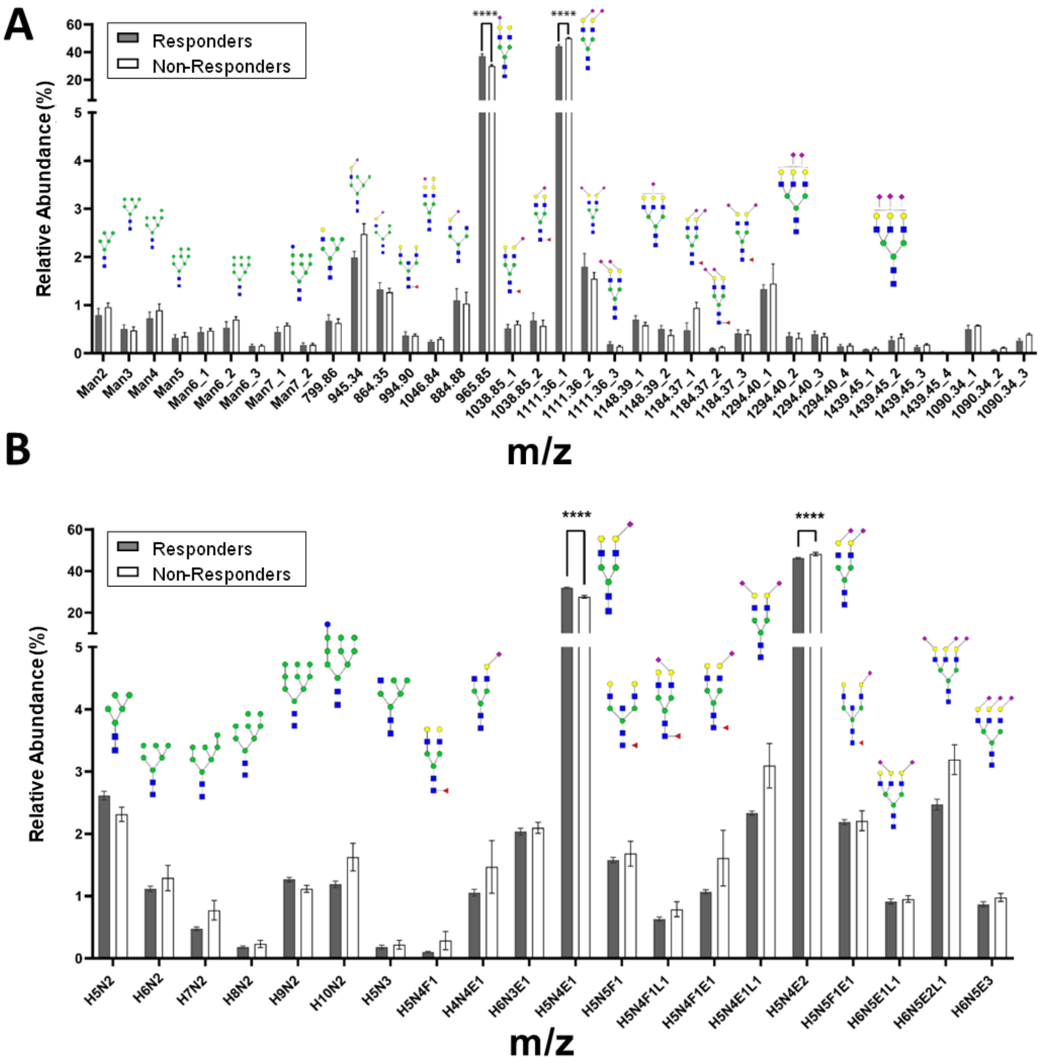

Figure S3. N-glycome of total chicken serum. (A) PGC LC ESI MS/MS: Individual sera from 10 responders and 5 non-responders were analysed. *m/z* of each N-glycan is specified as a function of its corresponding relative abundance (%). Isomers are represented as *m/z*_1, *m/z*_2 and so on. **(B)** MALDI TOF MS: Individual sera from 42 responders and 22 non-responders were analysed. Glycan composition used in the figure is explained as follows, H - Hexose, N - HexNAc, F – Fucose, L – α2-3 Neu5Ac sialic acid linkage, E – α2-6 Neu5Ac sialic acid linkage, N-glycan core – H3N2. Ethyl esterification gives distinct peaks for glycans containing α2-3 and α2-6 linked sialic acids. The corresponding glycan structures are drawn on top of the data points. Statistical significance was calculated using two-way ANOVA wherein the *p*-value was ≤ 0.0001.

Figure S4

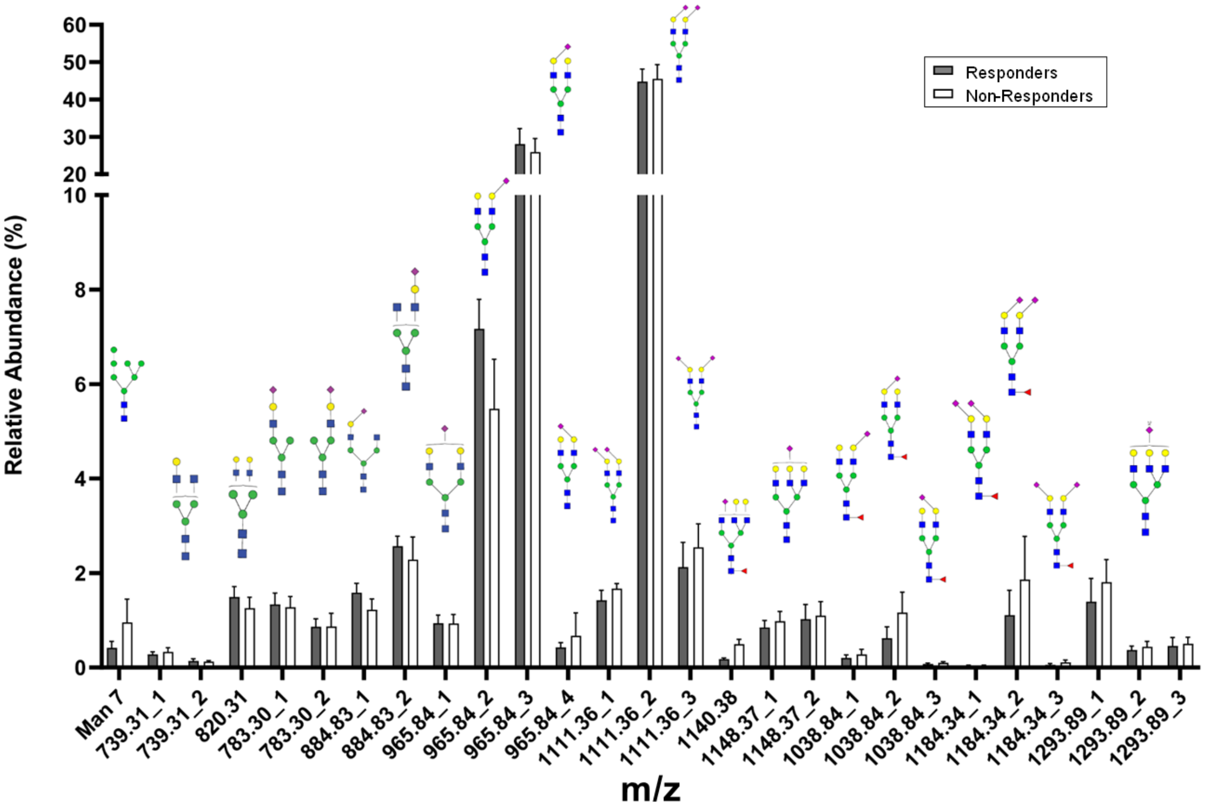

Figure S4. Comprehensive glycomics of chicken IgY using PGC ESI MS/MS. Purified IgYs from 4 responders and 5 non-responders were analysed. The corresponding glycan structures are drawn on top of the data points. The *m/z* of each N-glycan is specified as a function of its corresponding relative abundance (%). Isomers are represented as *m/z*_1, *m/z*_2 and so on. Statistical significance was calculated using two-way ANOVA wherein the p-value was ≤ 0.0001.

Figure S5

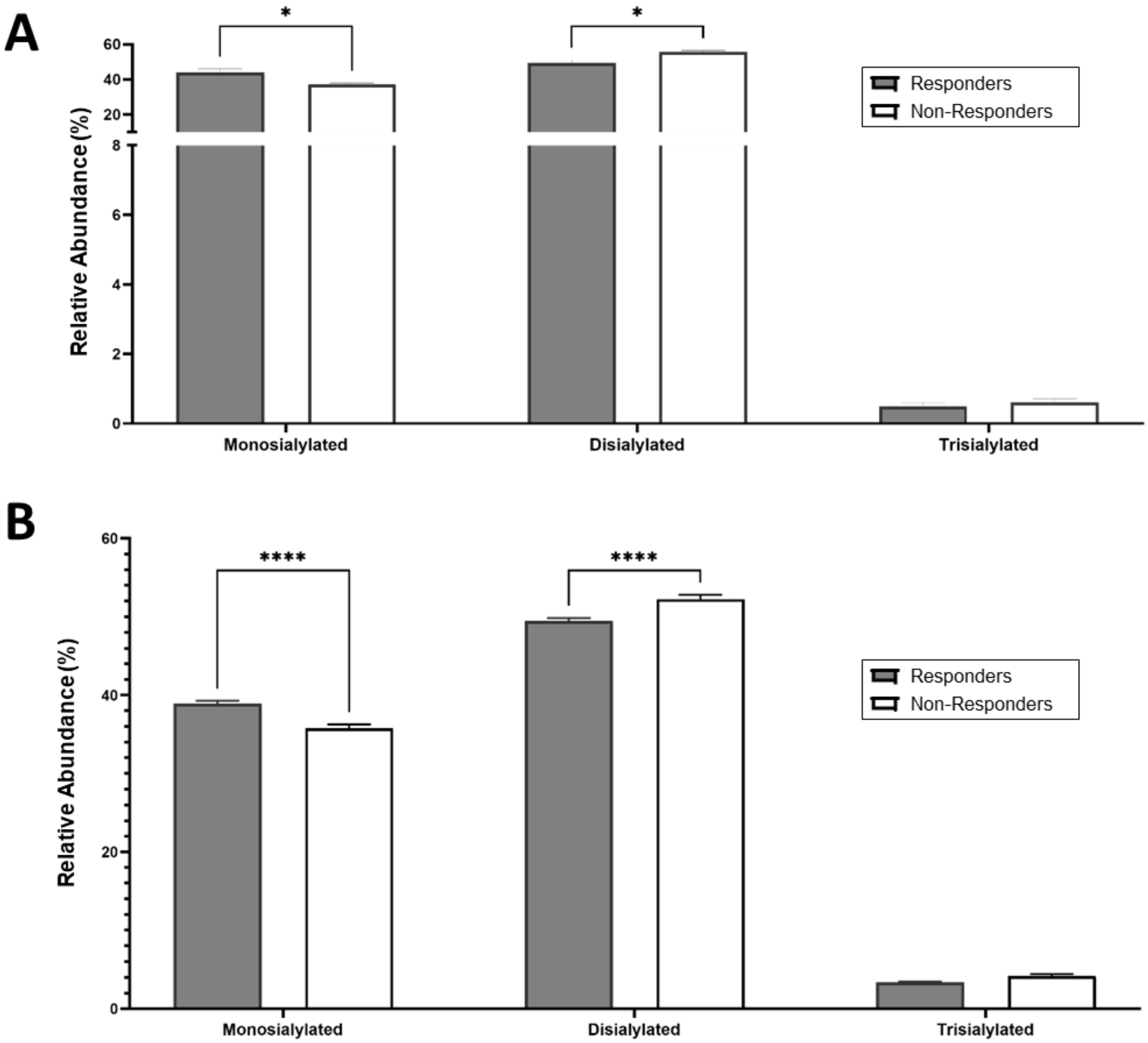

Figure S5. (A) Distribution of sialic acid-carrying N-glycans in total chicken serum analysed by PGC LC ESI MS/MS. Mono-, di- and tri-sialylated glycans in individual sera from 10 responders and 5 non-responders were analysed**.** Statistical significance was calculated using two-way ANOVA wherein the *p*-value was ≤ 0.0001. **(B) Distribution of sialic acid-carrying N-glycans in chicken serum analysed by MALDI TOF MS.** Mono-, di- and tri-sialylated glycans in individual sera from 42 responders and 22 non-responders were analysed. Statistical significance was calculated using two-way ANOVA wherein the *p*-value was ≤ 0.0001.

Figure S6

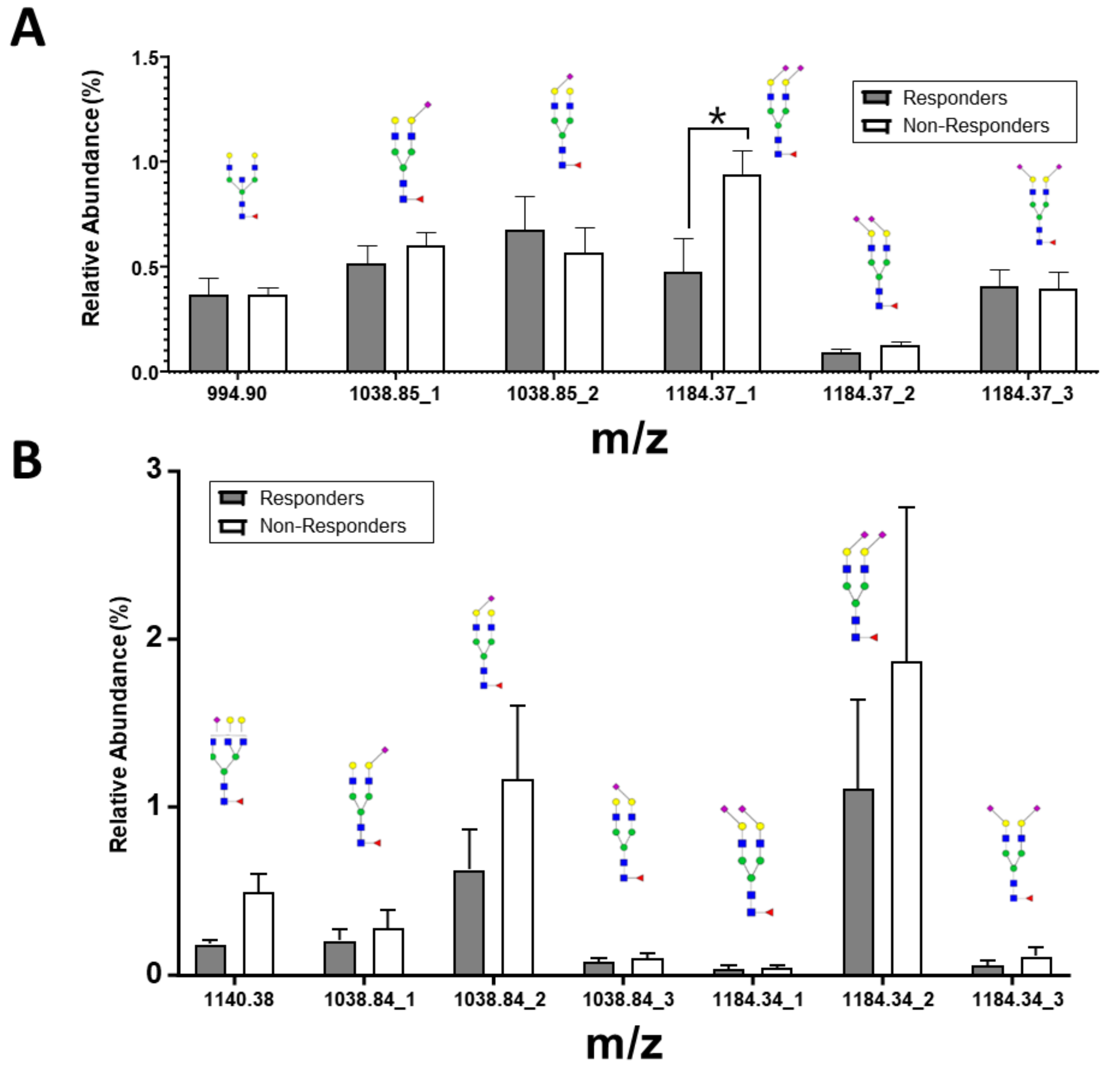

Figure S6. Distribution of core fucosylated N-glycans using PGC nanoLC ESI MS/MS. (A) Analysis of total chicken serum obtained from 10 responders and 5 non-responders. **(B)** Analysis of enriched chicken IgY glycans prepared from 4 responders and 5 non-responders. The corresponding glycan structures are drawn on top of the data points. The *m/z* of each N-glycan is specified as a function of its corresponding relative abundance (%). Isomers are represented as *m/z*_1, *m/z*_2 and so on. Statistical significance was calculated using two-way ANOVA, $p \leq 0.0001$.

Figure S7

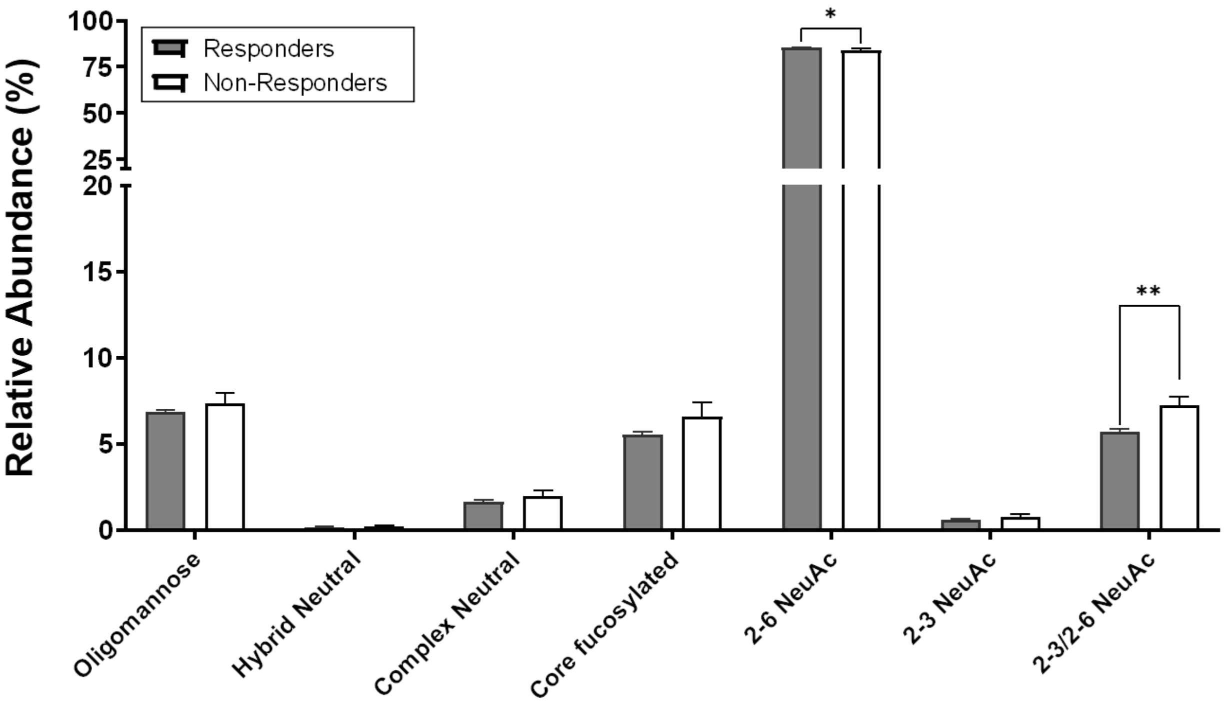

Figure S7. Distribution of N-glycans in chicken serum analysed using MALDI-TOF MS. Neutral, core fucosylated and 2-3 linked NeuAc carrying N-glycans in sera from 42 individual responders and 22 non-responder birds were analysed. Statistical significance was calculated using two-way ANOVA wherein the *p*-value was ≤ 0.0001.

Figure S8

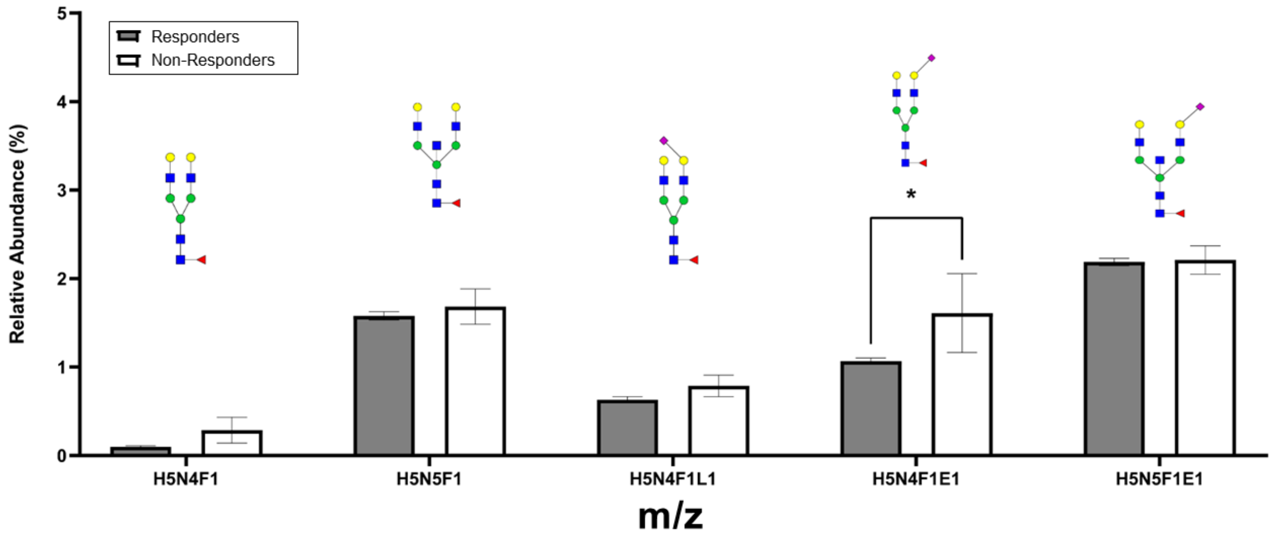

Figure S8. Distribution of core fucosylated N-glycans in the total chicken serum analyzed using MALDI TOF MS. The experiment consisted of 42 responders and 22 non-responders, respectively. Glycan composition used in the figure is explained as follows, H - Hexose, N - HexNAc, F – Fucose, L – α2-3 Neu5Ac sialic acid linkage, E – α2-6 Neu5Ac sialic acid linkage, N-glycan core – H3N2. Ethyl esterification gives distinct peaks for glycans containing α2-3 and α2-6 linked sialic acids. The corresponding glycan structures are drawn on top of the data points. Statistical significance was calculated using two-way ANOVA, $p \leq 0.0001$.

Figure S9

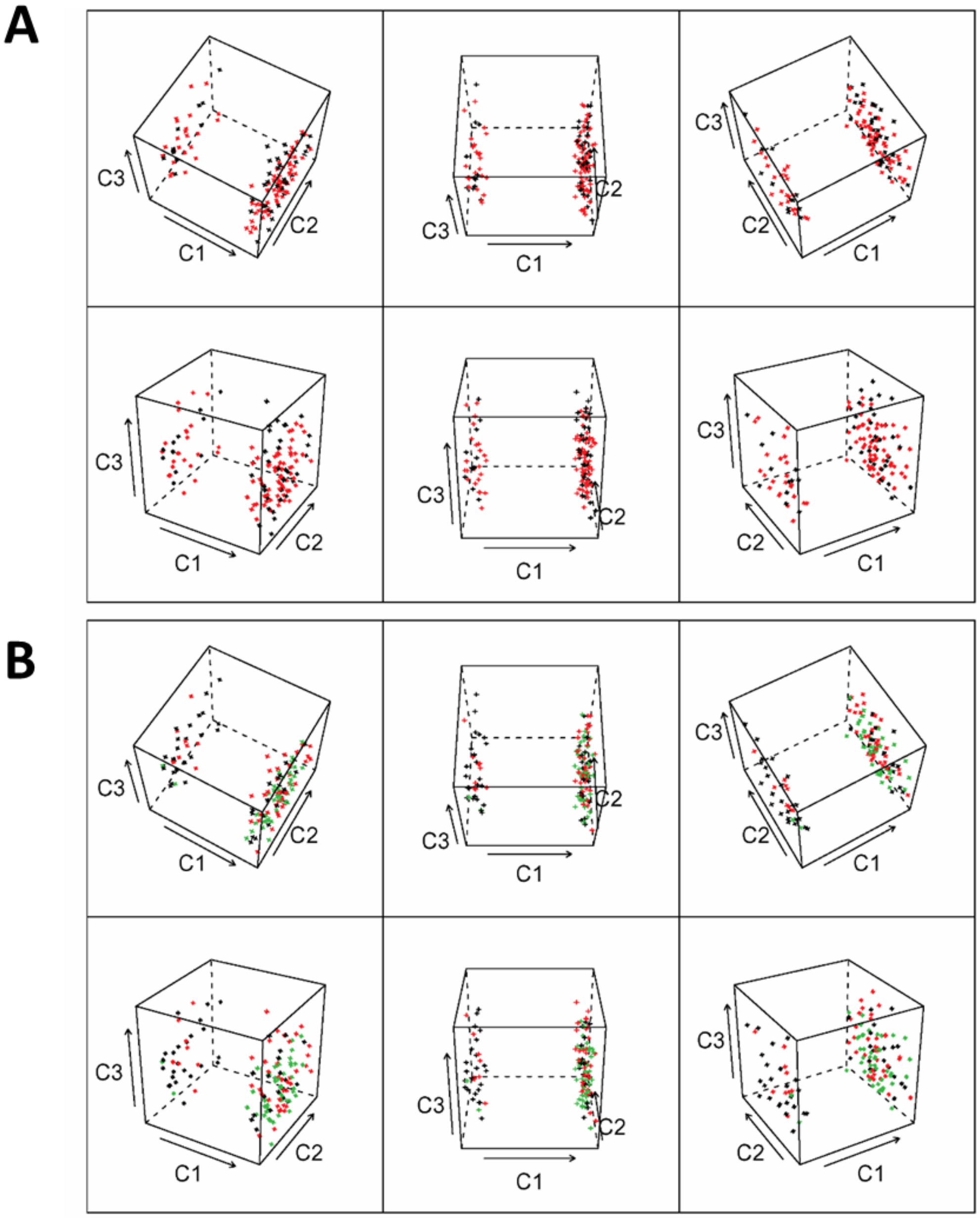

Figure S9. Chicken host phenotyping. Genome-wide association study (GWAS) results depicted by 3D-plots show the genetic distance between 134 chickens (88 responders and 46 non-responders). Each point represents one bird. Note that no DNA was obtained from bird no. 324, experiment C, non-responder, so that point is missing. **(A)** The points are coloured based on their group (black for non-responder and red for responder). **(B)** The points were coloured based on their batch (black, red, green for Experiment A, B, C, respectively). A chi-square test revealed no significant association between single-nucleotide polymorphisms (SNPs) and the responder versus non-responder phenotypes.

Figure S10

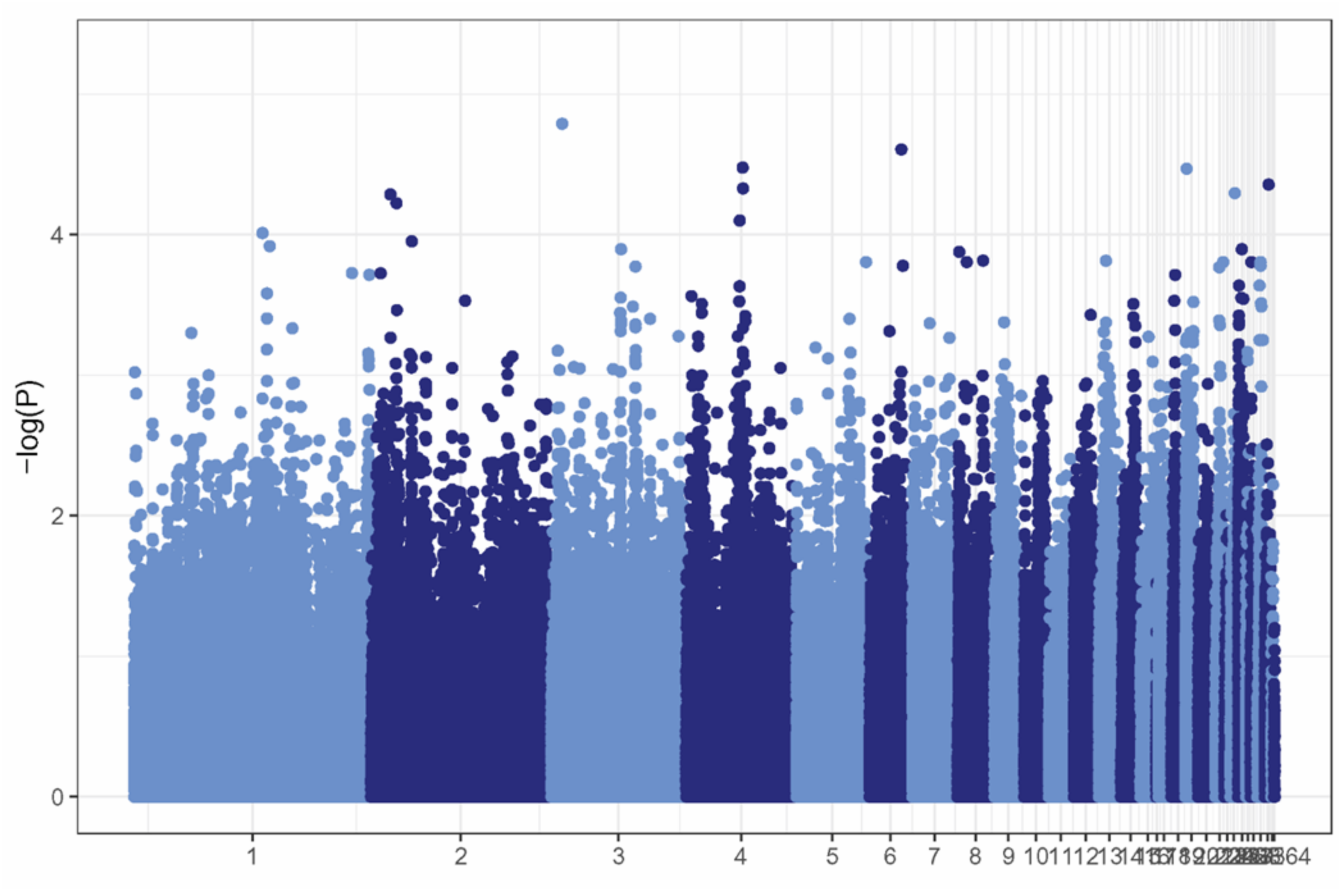

Figure S10. Manhattan plot of GWAS results. After false discovery rate (FDR) adjustment for multiple testing, no significant (FDR < 0.1) SNPs were found between responder and non-responder birds.

Figure S11

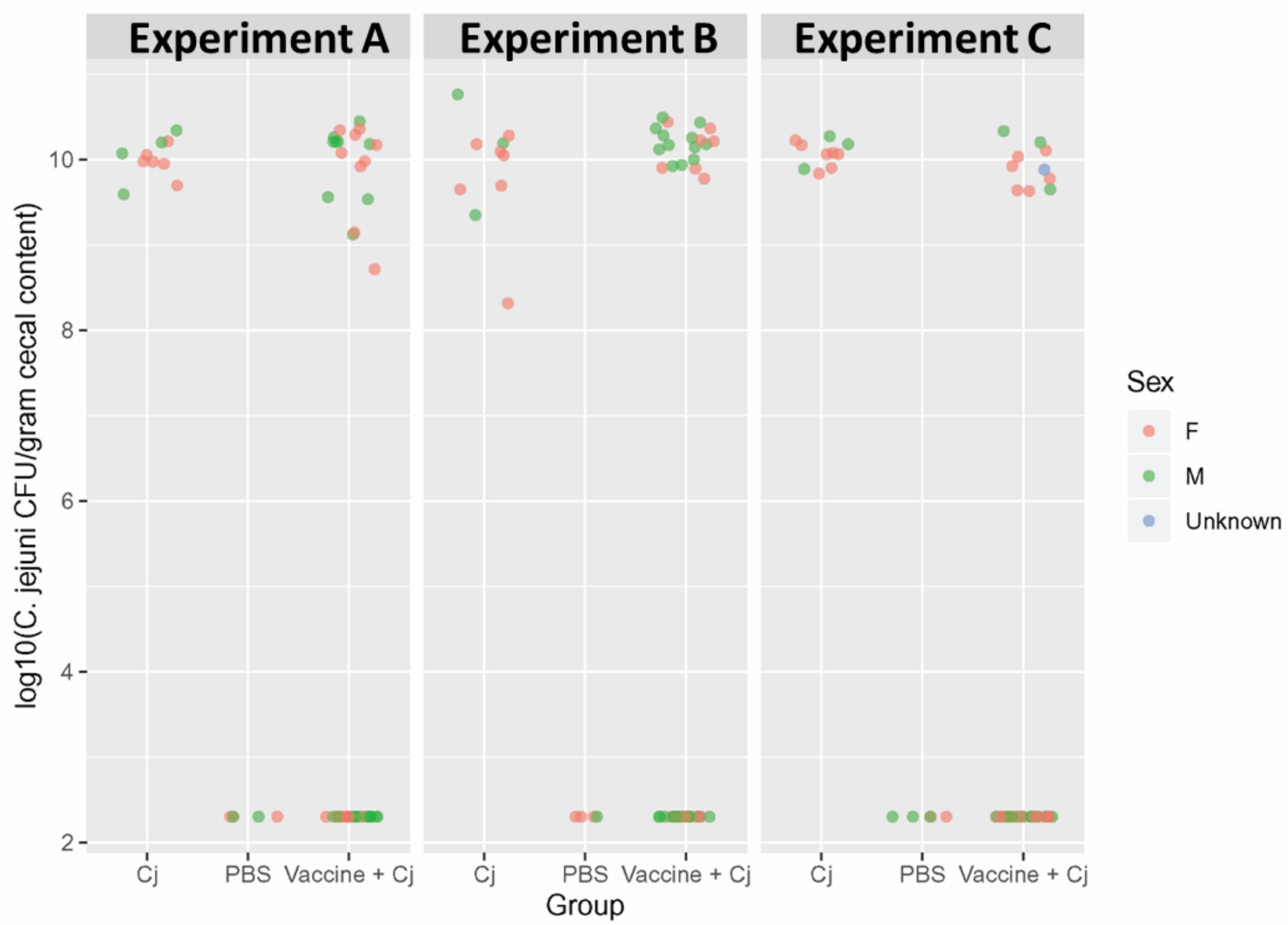

Figure S11. Sex of responder versus non-responder birds. Sexing results of 178 chickens (90 males (M, green dots)) and 88 females (F, red dots)) from vaccination Experiments A, B and C (indicated above each panel) based on their genotype of specific SNPs located on the W and Z chromosomes. Data were plotted against the colonization phenotype (expressed as log10 (*C. jejuni* CFU/gram cecal content)). No significant dependence was found between the response and sex by chi-square test (*p*=0.74). Individual groups are indicated below each panel.

Figure S12

A

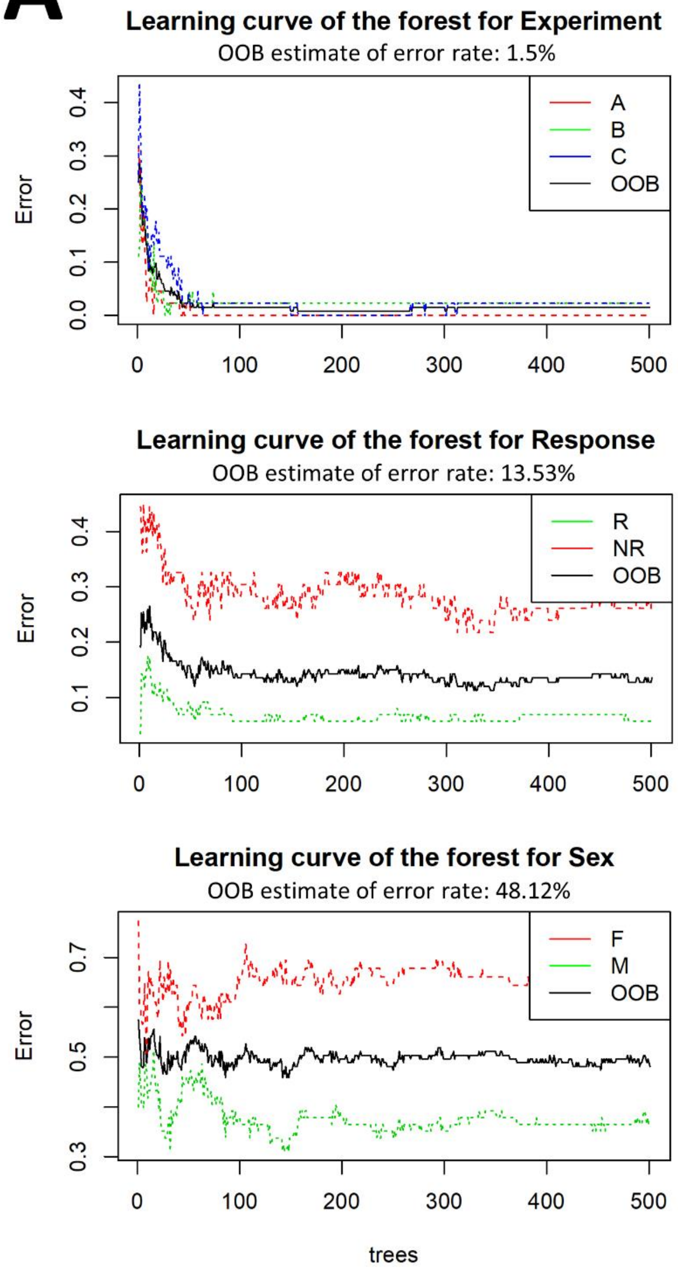
Learning curve of the forest for Experiment
OOB estimate of error rate: 1.5%
A
B
C
OOB
Error
0.0
0.1
0.2
0.3
0.4
0
100
200
300
400
500
Learning curve of the forest for Response
OOB estimate of error rate: 13.53%
R
NR
OOB
Error
0.1
0.2
0.3
0.4
0
100
200
300
400
500
Learning curve of the forest for Sex
OOB estimate of error rate: 48.12%
F
M
OOB
Error
0.3
0.5
0.7
0
100
200
300
400
500
trees

Figure S12 cont.

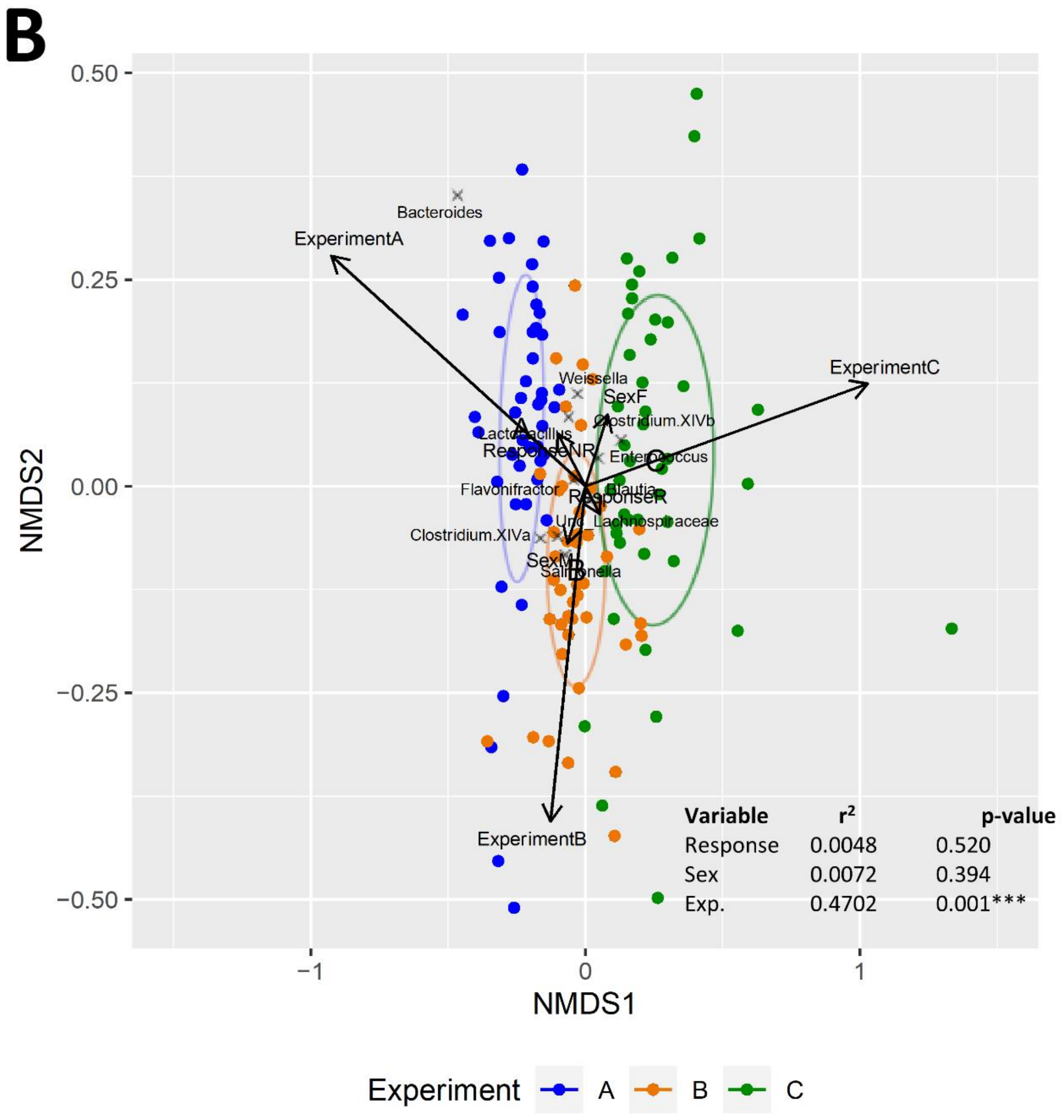

Figure S12 cont.

C

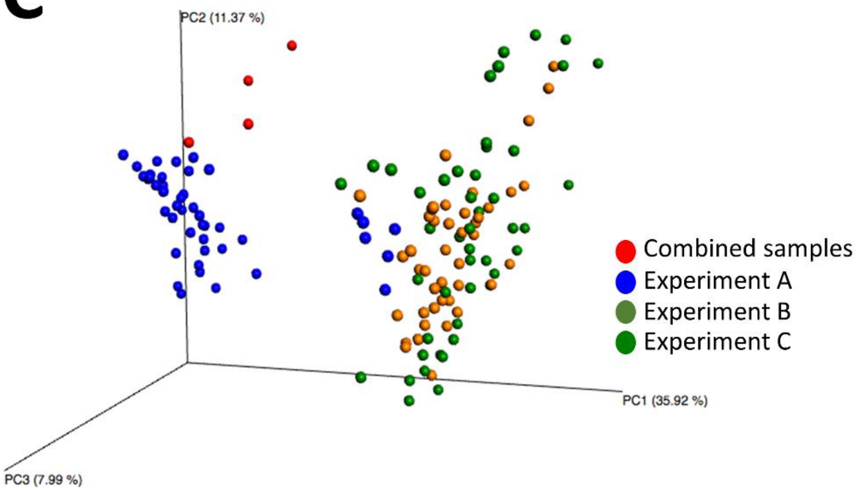

D

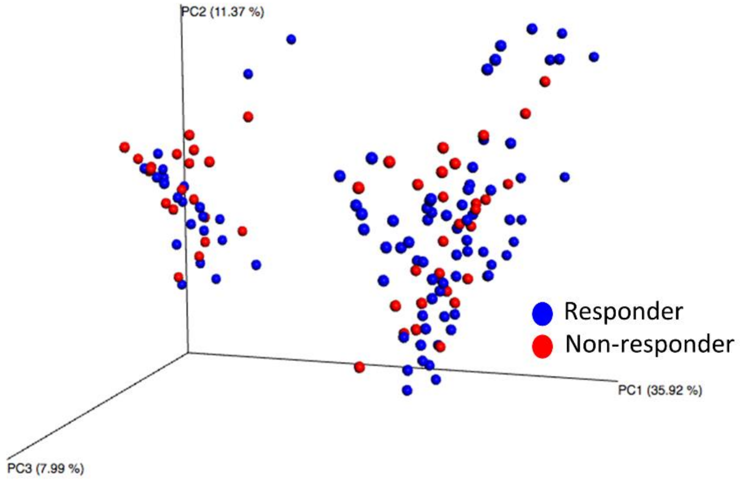

Figure S12. Random forest analysis, test of significance of supplementary variables in unconstrained ordination (Envfit function on NMDS) and principal coordinates analyses (PCoA). (A) Random forest analyses on abundance data were performed to determine the out of bag (OOB) estimate of error rate of the environmental variables under consideration. **(B)** Fitting of environmental factors in unconstrained ordination (Envfit function on NMDS) was used to determine the significance of the variables using permutation tests. The squared correlation coefficient (r^2) of the goodness of fit statistic determines the significance of the fitted factors. The length of the arrows indicates the strength of the correlation based on the r^2 value. The length of the arrows was amplified 4 times for visibility and clarity. Significance established at $p<0.05$. (C-D) Principal coordinates analyses of Euclidean distances show clustering of samples by **(C)** experiment and by **(D)** vaccine response. R, responder; NR, non-responder, F, female; M, male.

Figure S13

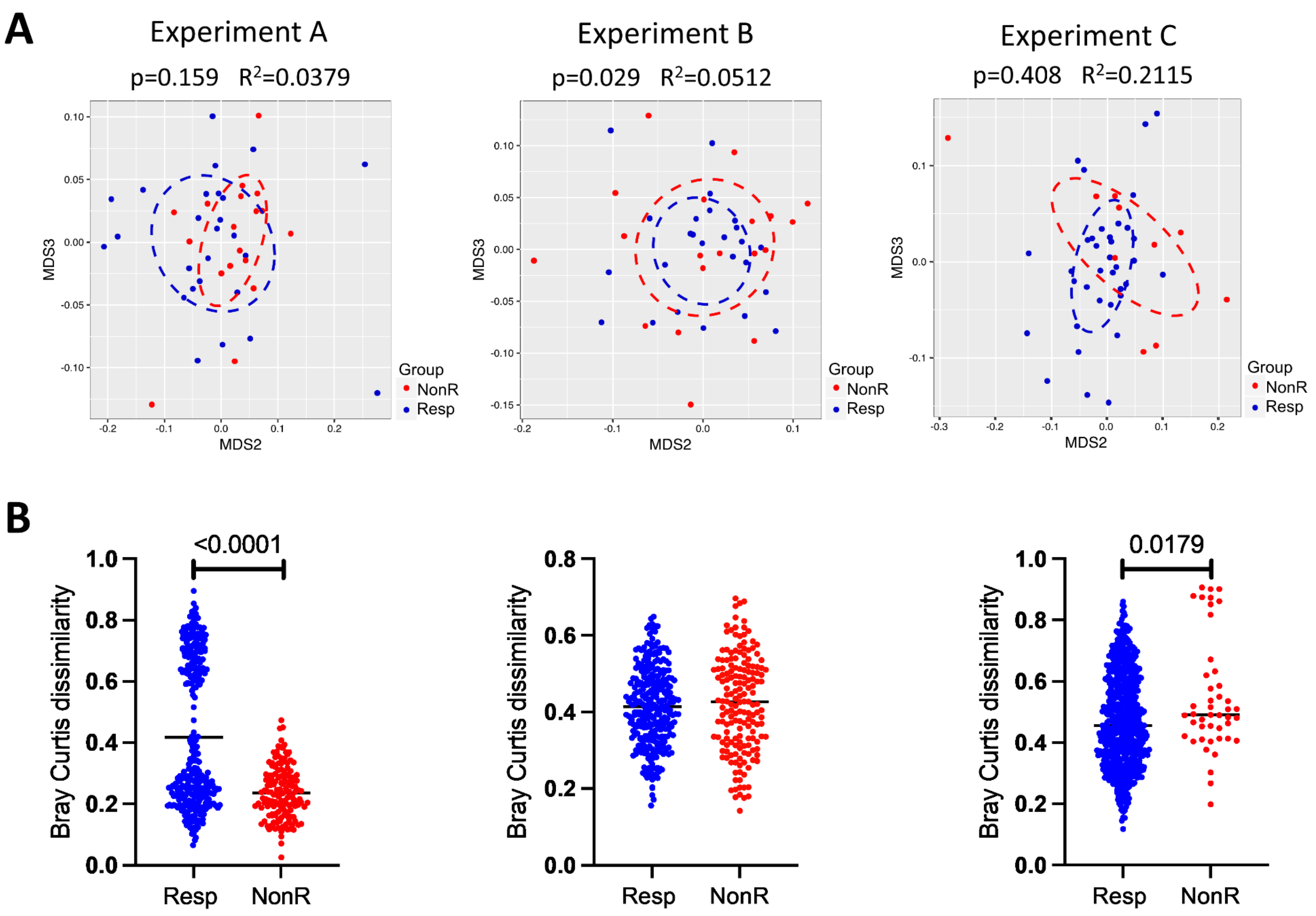
A
Experiment A
p=0.159 R2=0.0379
Experiment B
p=0.029 R2=0.0512
Experiment C
p=0.408 R2=0.2115
MDS3
MDS2
Group
NonR
Resp
B
Bray Curtis dissimilarity
<0.0001
0.0179
Resp
NonR

Figure S13 cont.

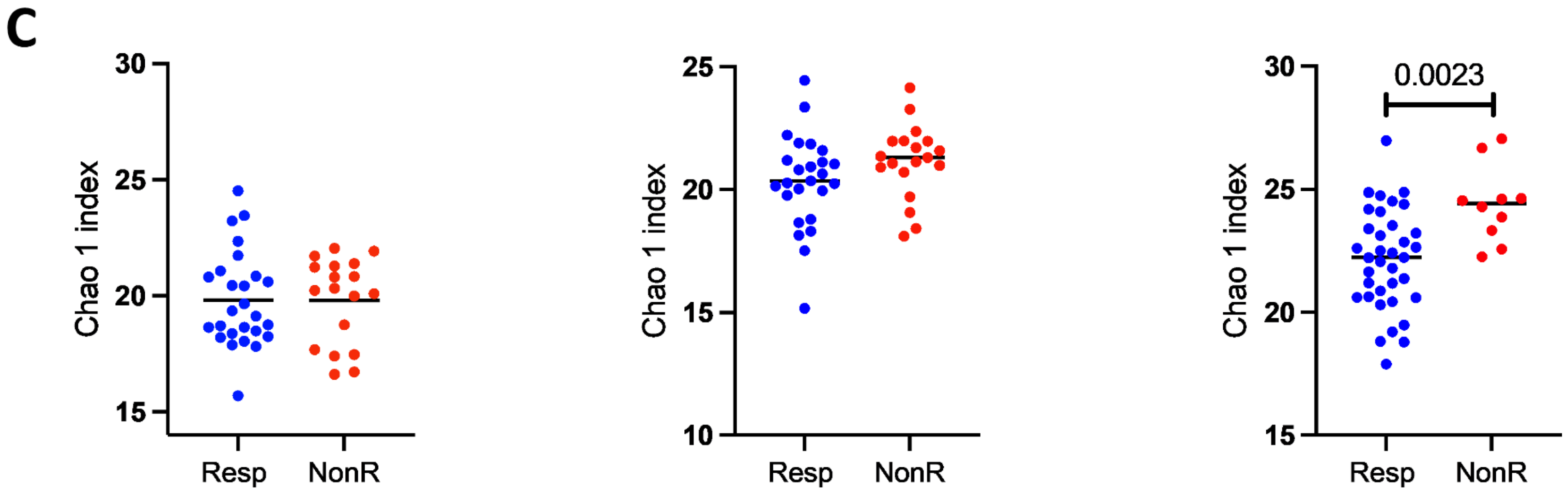

Figure S13. Diversity analysis between responders and non-responders for each vaccine experiment. (A) NMDS plots based on Bray-Curtis distances analyzed by PERMANOVA. **(B)** Scatter dot plots of Bray-Curtis dissimilarities analyzed using the Mann-Whitney test (Wilcoxon rank-sum test) to determine differences in β-diversity. **(C)** Scatter dots plots of Chao 1 indexes analyzed using the Mann-Whitney tests to determine changes in α-diversity. OTUs were used for all diversity analyses. Lines represent median values. Significance was considered at $p<0.05$.

Figure S14

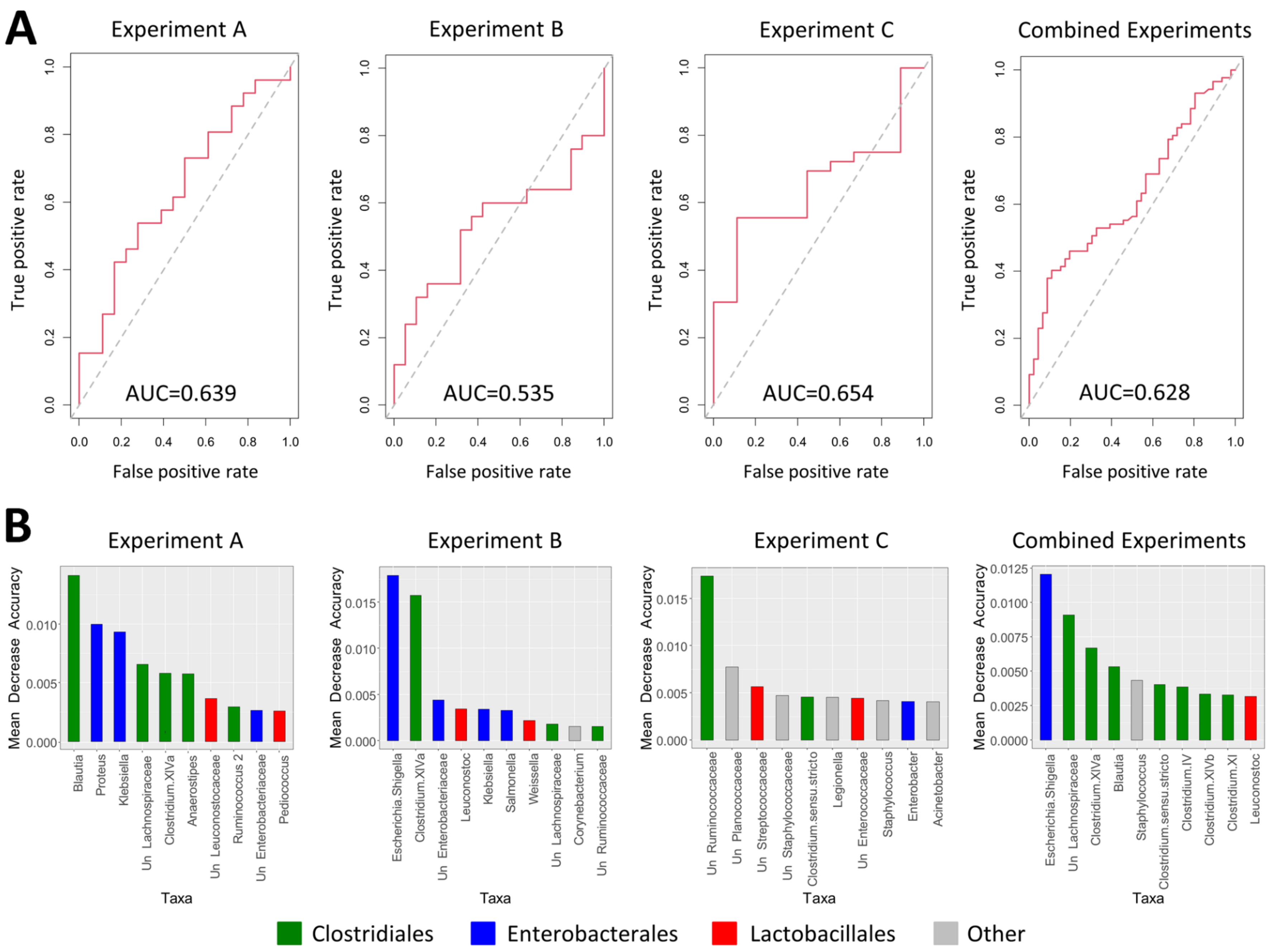

Figure S14. (A) ROC plots (using genera and excluding *Campylobacter* from the equation) were generated for the response factor and the area under the curve (AUC) was calculated for individual experiments and for all experiments combined using the ROCR package in R statistical software. **(C) Barplots of variance importance per experiment** as determined by mean decrease accuracy as part of random forest analyses. Un, unclassified.
